# Global language geography and language history: challenges and opportunities

**DOI:** 10.12688/openreseurope.18421.1

**Published:** 2024-10-07

**Authors:** Matthias Urban

**Affiliations:** 1 Dynamique Du Langage, Centre Nacional de la Recherche Scientifique, Lyon, France; 2 Universite de Lyon, Lyon, France

**Keywords:** Language geography, language dispersal, linguistic diversity, typology, historical linguistics

## Abstract

As the several thousand languages spoken by people all around the world became more and more systematically assessed and catalogued in the 20th century, it became clear that linguistic diversity is unevently distributed across the globe. Up to the present day, the reasons for that are poorly understood. Linguists are thus in the embarassing situation that they do not understand significant regularities in the way the objects of their study –languages– pattern; human sciences at large are faced with the fact that the way humans produce that key cultural product which is often seen as defining the essence of what makes them humans –language–remains in the dark. In this essay, I explore three interrelated strands of thought associated with the problem of explaining patterns in global language diversity to create a perspective that is different from those explored so far. First, I suggest that instead of looking at present-day levels of diversity and find parameters of variation between the regions in which they are spoken, we should take a process-based approach that looks into how these distributions were generated. Related to this point and in contradistinction to extant work, second, I advocate an inductive approach that departs from qualitative case studies which inform theory-building. Third, I ponder that, in contrast to the traditional focus of historical linguistics on language diversification and expansion, understanding how the ranges of languages are reduced might be the key missing piece of evidence in a global theory of language diversity and its genesis.

## Plain language summary

This essay reflects on the distribution of linguistic diversity across the globe, which at present cannot be explained satisfactorily. I suggest that to understand this distribution we need to look at actual, concrete processes that have generated the ranges of individual languages, and that we can extrapolate from these to understand patterns in linguistic diversity at large.

## Introduction: the puzzle of linguistic diversity

During the 19th and 20th century, our knowledge concerning the diversity of human languages has increased massively. We know now that there were, and still are, many more distinct languages spoken around the world than what has generally been thought possible some centuries ago. We also know now that the way these languages function is massively more diverse than has been thought possible as well ( Evans & Levinson, 2009).

“Language diversity” can mean several related things that must be distinguished carefully. First, it can refer to the sheer number of languages relative to the size of a study area, e.g. a country (this is sometimes called “language richness”). Second, “language diversity” can reference the number of different language *families* relative to the size of a study area (this is sometimes referred to as “phylogenetic diversity”). These two parameters often, but not always, correlate. In Africa, for instance, the sub-Saharan belt hosts hundreds of clearly distinct languages, but they all belong to the so-called Bantu branch of the Niger-Congo family that began to diversify several thousand years ago; these languages are also relatively uniform in how their grammars work. This takes me back to the observation of typological diversity: orthogonally to these two dimensions, the languages in a given study area may also be very similar or very diverse in how they sound and how their grammars work (this parameter is sometimes also called “structural diversity”, Nettle, 1998). Northern Eurasia scores relatively low on all there counts: there are relatively few different languages; these belong to a small number of distinct language families; and most, in particular those resulting from recent language spreads, are quite similar in how they sound and how their grammars work.

Some regions in particular have turned out to be massively diverse linguistically. A textbook case is the island of New Guinea, which alone hosts almost 1000 distinct languages. Given the size of the island, this means that an observer will in some cases encounter a different language as they just travel to the next village. Although New Guinea is an extreme case, other parts of the world are likewise hyperdiverse. One such region is South America, especially the forested lowlands of greater Amazonia; yet another such region would be California before Europeans colonized the region (which highlights that the diversity is massively threatened, and, in spite of successful efforts at lange revitalization, we must reckon with a further loss in diversity – see Krauss, 1992).

**Figure 1.**
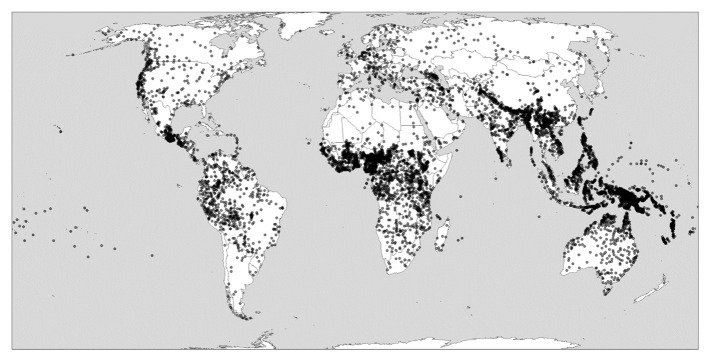
Global linguistic diversity (language richness). Created using the R package glottospace ( Norder *et al.*, 2022).

Figure 1 shows such hotspots of linguistic diversity (with each dot representing a distinct languages) while also demonstrating how other regions are much less diverse.

**Figure 2.**
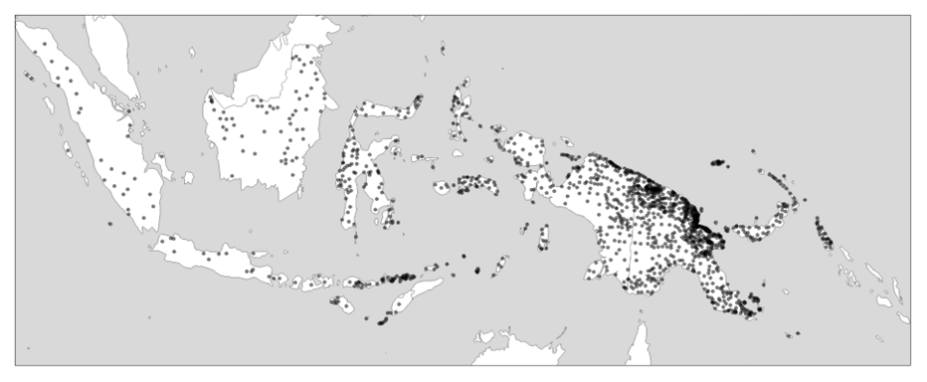
Linguistic diversity (language richness) in Indonesia and Papua New Guinea Created using the R package glottospace ( Norder *et al.*, 2022).

This pattern of differential diversity is not only observed within continent or island-sized areas such as those I have just mentioned, but scales down to variation *within* such areas: In New Guinea, it is the coastal areas in the north of the island that host disproportionaly much of the island’s linguistic diversity, while the New Guinea highlands, which traverse the island longitudinally, are lower in diversity both measured in terms of individual languages and in terms of different families. ^1^ Figure 2 gives an impression of this.

**Figure 3.**
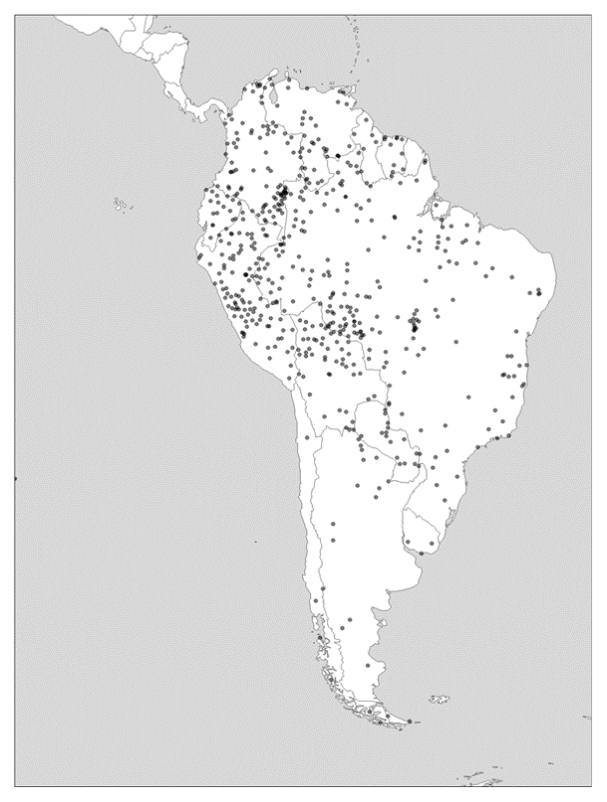
Linguistic diversity (language richness) in South America. Created using the R package glottospace ( Norder *et al.*, 2022).

As can be observed in Figure 3, in South America, diversity levels in greater Amazonia, while high everywhere, are particularly pronounced on the eastern margins of the lowlands just before the land begins to rise slowly. In the Andes themselves, and as one moves southward to Patagonia and Tierra del Fuego, diversity levels become notably lower. In North America it is only California that boasted a hyperdiverse mosaic of languages or language families, whereas to the east of the Rockies, diversity is measurably lower.

It is such observations of nested diversity clines that suggest that it is no mere coincidence of history that New Guinea, Amazonia, and California are hyperdiverse, whereas Greenland, Patagonia, and eastern north America are not to the same extent. However, up to the present day, the reasons for regionally uneven diversity levels are not understood.

As the differential patterning of linguistic diversity has come to the attention of linguists and scholars in other disciplines, it has been observed that there is a striking congruence between linguistic and biological diversity ( Gorenflo *et al.*, 2012; Maffi, 2005; Moore *et al.*, 2002). Also here, the relationship to some extent obtains at various scales (though at some levels of resolution, it breaks down: Manne, 2002). For instance, in South America, the Amazonian fringe and the cloud forest ecotone at the intersection of Andes and the eastern lowlands are not only hotspots of linguistic, but also biological diversity.

Languages are cultural products, shaped by the communicative behaviour of their speakers ( Du Bois, 1987) and the social ecologies they are embedded in ( Pakendorf *et al.*, 2021; see more extended discussion in the following section). They are *not* species; and it goes without saying that neither are their speakers which therefore we could or should not study as if they were. Drawing an analogy between biological evolution and “cultural evolution” is as tempting as potentially dangerous and simplifying, especially as we have a lot of qualitative knowledge on the maintenance of linguistic diversity and their social ecologies that are specifically human (again, more extended discussion is in the following sections). Still, undeniably, languages cover geographical space in ways that are strikingly similar to the way to biological species.

Like Hua *et al.* (2019), I tend to view this association as ephemeral (linguistic and biological diversity co-vary with climate and/or environment, which shapes the distribution of both) rather than causal (humans diversify culturally in areas with high biodiversity more readily because of the richness of available resources).

The latter scenario would be consistent with a particular interpretation of a prominent hypothesis that has been suggested to explain the uneven linguistic diversity of the world. In the next section, I turn to work that investigates linguistic diversity on a global level, including this hypothesis of “ecological risk”. I also discuss some of the issues I see with extant work, and questions that still remain open, either because studies so far have yielded contradictory results or because they have not been a focus of attention.

## Current perspectives (and their problems)

There are, at the very least, three dimensions to the problem of explaining global linguistic diversity: first, there is the question which climatic and environmental factors (if any) shape and lowbeds linguistic landscapes; second, there is the question if the congruence between cultural and biological diversity is a coincidence, and, if not, how it should be explained; and third, especially because languages emphatically are not species, there is the question how such factors, if they could be identified, link up with actual human linguistic and non-linguistic behavior.

In the last 25 years or so, a number of studies have appeared that adress the general question of linguistic diversity and its drivers. However, they have mainly focussed on the first dimension, while the actual behaviour of people that, in the long term, could lead to these distributions has received much less attention, a point to which I will return later.

Early studies ( Cashdan, 2001; Collard & Foley, 2002; Hillebrand, 2004; Mace & Pagel, 1995) mainly noted a latitudinal gradient to global levels of linguistic diversity (see also Gavin & Stepp, 2014).

When it comes to more concrete factors and possible explanations, Nettle ( 1996; 1998) was an influential pioneer, and his “ecological risk” hypothesis is still viable today: Nettle argued that lingutisic diversity correlates not just with latitude, but more directly with the length of the Mean Growing Season, i.e. the number of months per year that allow, given further local conditions like temperature, rainfall, etc., vegetation to grow. The length of the Mean Growing Season, in turn, is taken by Nettle as a proxy for “ecological risk”: Where it is long, societies are hypothesized to be self-sufficient: the environment allows enough resources for most parts of the year to ensure reliable subsistence. Where climatic conditions are less stable, or where resources are scarcer due to a shorter Mean Growing Season, groups must increasingly derisk their subsistence base. This involves the explotation of resources of larger tracts of land, and/or the maintenance of stronger inter-community ties and larger networks – hence larger “ethnolinguistic groups”. This should in principle be relevant both for agrictulturalist and more “traditional” lifestyles involving hunting and gathering, as ultimately plants form the basis for subsistence in both cases.

Nettle’s (1998) studies had some analytical problems. In the meantime, the size of the data analyzed; the number of possible environmental variables considered; and in general the sophistication of relevant work has grown enormously. For instance, it has been shown that spatial autocorrelation, a typical phenomenon in spatially structured data, can confound results in research on the environmental and social conditioning factors of linguistic diversity ( Cardillo *et al.*, 2015). It has also been noted that the effect of surveyed climatic variables may be non-stationary and interact in locally specific ways in generating linguistic diversity or inhibiting it ( Pacheco Coelho *et al.*, 2019). As a whole, research, reassuringly, shows the typical signs of maturation of a field of scientific investigation.

At the same time, however, the results of the relevant studies still differ widely. We have now a whole series of articles, often in high-standing journals and using sophisticated methodology, that share the general concern of identifying ecological drivers of linguistic diversity. However, the results are still as contradictory as noted ten years ago by Gavin *et al.* (2013). For instance, Sutherland (2003) finds no evidence for Nettle's ecological risk hypothesis. Axelsen and Manrubia (2014) identify the presence of rivers and terrain roughness (technically, “rugosity”) as factors that are conducive to the rise of linguistically diverse landscapes – but earlier Currie and Mace (2009) only found a weak effect of rugosity (which they measured differently, however). Hua *et al.* (2019), in turn, found these to be relatively negligible compared to climate and year round productivity, consistent with an account like that of Nettle. and Derungs *et al.* (2018) interpret their findings as likewise consistent with the “ecological risk” hypothesis. Then again, Derungs *et al.* (2018) concluded that climate –latitude, precipiation, and temperature – is more relevant for linguistic diversity of food-producing (agriculturalist) societies than for hunter-gatherers, which would mean that “ecological risk” should affect different types of people in diferent ways.

I now turn to the crucial question of how climatic and environmental factors, if identifiable, could lead to actual observable linguistic and non-linguistic behavior of people that, in turn, set into motion processes that would generate observed patterns of diversity. As sketched above, an answer to this question is suggsted by Nettle in the “ecological risk” hypothesis. However, as far as I am aware, what effect, if any, ecological risk has on language diversity has so far never been shown through any detailed qualitative case study. Notably, in direct contradiction to the idea that ecological risk fosters a smaller number of languages with wider ranges, in traditional settings, it is precisely linguistic diversity which, sustained in multilingual landscapes, can be a strategy "that maximizes alliances and protective networks through different languages“ ( Lüpke, 2016: 53). Where case studies on the ecology of linguistic diversity in the tradition of the ethnography of communication ( Hymes, 1964) exist, it usually turns out that it is language ideologies, of one kind or another, that sustain stable situations of linguistic diversity ( Pakendorf *et al.*, 2021). For instance, in the Vaupes region of the Amazon, linguistic exogamy together with a linguistic ideology that deprecates language mixing creates a situation in which a range of distinct languages belonging to different language families are maintained and converge with each other grammatically, but not lexically (e.g. Epps, 2020). Also some big data quantiative studies –such as that by Antunes *et al.* (2020) on New Guinea– suggest that environmental factors alone are insufficient to explain observed levels of linguistic diversity, and that the vicissitudes of human population dynamics, but also socio-cultural factors, must be taken into account.

Qualitative, “thick” ( Geertz, 1973) descriptions of language ecologies, furthermore, suggest that notions like “group boundary formation” ( Gavin *et al.*, 2013), i.e. the use of a certain language to identify one’s “ethnic group”, set it off against other such groups, and thus yield “ethnolinguistic groups” are far from universal. The notion “ethnolinguistic group”, incidentally, has mostly been abandoned by anthropologists, and seems to be mainly used today by “outsiders” such as linguists, geographers, etc. The “ethnolinguistic group” is an idealized fiction that brings to mind Romanticist equations between “people” and “language”, which are inadequate for describing language use in many parts of the world. Nettle himself, who relies on the “ethnolinguistic group” as a unit of analysis, quotes Brooks (1993: 27) to the effect that in West Africa, “individuals and families change their language and modify their social and cultural practices in ways that are often perplexing to outsiders.” In other words, language is not firmly linked to particular individuals that together would make up an “ethnolinguistic group”, but language use is dependent on social context, roles, and local language ideologies. An influential analysis of such configurations in the Balkans is in Irvine and Gal (2000); for the ancient and present-day Central Andes in Babel (2018) and Urban ( 2018; To appear); and for Upland Southeast Asia and the Himalayas in Scott (2009) and Shneiderman (2010). As Evans (2018: 13) aptly puts it, “the focus on language diversity as something manifested by discrete, internally coherent entities can remove the very types of evidence we need to tackle the diversification problem.” On the other hand, there *are* qualitative studies that suggest that something akin to “group boundary formation” is actually going in some parts of the world not just in the context of modern national states, but also in traditional societies. Such essentializing roles of language(s) are found, also in traditional “small scale” societies, e.g. in linguistically diverse areas of Papua New Guinea (e.g. Laycock, 1982; Sankoff, 1980). In sum, the qualitative literature clearly suggests that the recruitment of languages as group markers are far from universal, which suggests that general, one-size-fits-all explanations for the genesis of linguistic diversity may be problematic. Such examples, however, only show that language ideologies can *sustain* stable situations of multilingualism in regions of high linguistic diversity. They do not show how language diversity is *generated* in the first place. On the one hand, the answer to this question is rather simple, as historical linguists have a very good understanding of how language change works. However, when and why it occurs – Weinreich *et al.*‘s (1968) actuation problem– is much less clear. However, once again, a wide range of sociolinguistic studies suggests that the answer has to do with the local social dynamics of linguistic interaction (at least in modern urbanized societies such as those where English is spoken).

Lest I be misunderstood, my point is not that ecological risk, or any other climate-related notion, does not have an influence, however indirect, on what parts of the world stabilize at what level of linguistic diversity. It is rather obvious that there *must* be some such proximate or, more likely, ultimate effect, even though it remains very elusive. I merely wish to point attention to the fact that we have not, again as far as I am aware, observed climate- and environment-related factors “in action” that would generate linguistic diversity in a set of diverse context (such as those sketched above) and that would be capable to explain language diversity globally. According to Evans (2018) who, presents the most complete model for how all these factors might link up, climatic conditions *do* shape societies, their values, and their attitudes towards others, and these in turn govern linguistic behaviour in conversational praxis within one’s own social group and with outsiders. From such accounts, it becomes clear that how climate and environment shape linguistic diversity is a significant more complex problem than what much of the extant literature assumes.

In sum, at present, research is in a remarkably open situation. The questions are on the table, but I think it is fair to say that answers that are robust to different analytic approaches and datasets and that are consistent with actually observed linguistic and non-linguistic behaviour have not crystallized yet. The impasse pertains most pressingly to the first two dimensions of linguistic diversity that I have sketched in the introduction. Astonishingly, linguists do not understand the ways in which their objects of study –languages–- are distributed on the largest imaginable scale, and especially why they are distributed this way. This is in stark contrast to the micro-levels of variation. Dialectologists, since the 19th century, have developed methods to describe and (at least to some extent) explain how features of pronunciation, grammar, and words change in geographical space, and the entire discipline of variationist sociolinguistics explores how language varies in social space. It is also in contrast to what we, as linguists, know on the distribution of features across whole languages and large regions (a field of study often called “areal typology”, in which clines and very large skewings, not dissimilar to those concerning language diversity, have been observed up to continental scales – Bickel, 2020; Dryer, 1989; Güldemann & Hammarström, 2020; Nichols, 1992; Urban *et al.*, 2019). Zooming out from linguistics, the situation is also relevant to the human sciences at large, which are faced with the fact that the processes by which human societies, within and between each other, produce that key cultural products which they have used to define themselves since antiquity –languages–- remains in the dark.

## Dynamizing linguistic diversity

As the main contribution of this essay, in this section I explore a line of reasoning that might lead to perspectives on language diversity and its origins that is able to bring together the abovementioned perspectives more satisfactorily. Since this is an essay, my discussion is mainly programmatic; whether the picture I will try to get into focus has merit is an open question. I mainly want to make three interrelated points: first I suggest that instead of attempting to find parameters of variation (climatic, environmental, political, etc.) between regions with differential levels of linguistic diversity, a process-based approach that looks into how these distributions were generated can furnish new perspectives that would be otherwise missed. In this vein, I suggest a way to dynamize the question of linguistic diversity and its drivers in a way that at the same time references the observed congruence between linguistic and biological diversity. Second, I ponder that, in contrast to the traditional focus of historical linguistics on language diversification and expansion, understanding how the ranges of languages are reduced might be the key missing piece of evidence in a global theory of language diversity and its genesis. Related to this point and in contradistinction to extant work, third, I advocate an inductive approach that departs from qualitative case studies which inform theory-building.

The starting point for developing my argument is the well-known distinction between spread and residual (or accretion) zones. Nichols ( 1992; 1997) distinguishes these as prototypical and contrasting types of language distributions in geographical space, and sketches their underlying dynamics. The dichotomy crucially references the same uneven distribution of linguistic diversity, that has come to the fore since 20 years or so at the interface of language, environment, and ecology, and at the same time contains elements of a theory to account for these dynamically.

As far as language geography is concerned (there are further more properly linguistic characteristics), spread zones, in the definition of Nichols (1992), are dominated by relatively few language families (i.e. have low phylogenetic diversity), and at times even just contain individual languages (i.e. low language richness). Relatedly, language distributions in spread zones are shaped by frequent and long-distance language expansions, which tend to completely or almost completely obliterate preexisting languages. These may include the languages that spread via earlier episodes of such expansions (“spread-over-spread” dynamics). As is expected in situations of rapid and long-range language spreads, the spreading languages are quite similar to one another as a result of the little time available for diversification through language change (i.e. low typological diversity). Thus, in spread zones, which languages and language families dominate the linguistic landscape can change drastically and quickly, but net linguistic diversity does not: it remains low on all three counts.

Residual zones, in the definition of Nichols (1992: 14–15) have the following characteristics: they contain old families (i.e. ones that are deeply diversified internally – this does not necessarily mean that these families must contain many individual languages, but that the languages belonging to its different branches are not closely related, which indicates a long time of internal differentiation). “Old families” in the relevant sense should be taken to include language isolates, which are the sole representative of lineages that are so old that relatives cannot be detected anymore. No major language expansions originate from residual zones, but they may attract instrusive languages and thus serve as a linguistic “refugium of sorts” in Nichols’s words). The arrival of these languages, however, do not lead to significant levelling of preexisting linguistic diversity in the residual zone, but rather, in addition to processes of diversification through language change that take place relatively undisturbedly, contribute further to a residual zone’s linguistic diversity.

Nichols’s prototypical residual zone is the Caucasus, which hosts the old Nakh-Daghestanian and Kartvelian language families. These language families probably originated *in situ* rather than somewhere else, and seem to have differentiated within the area without having been ousted by language spreads like those that are characteristic of spread zones (see Nichols, 2004 for further details on Caucasus-internal language dynamics). In addition to what appears to be largely *in situ* diversification of autochthonous linguistic lineages, representatives of larger language families of the steppe, Indo-European (Ossetic) and Turkic languages (e.g. Kumyk) have added themselves to the autochthonous diversity.

Nichols also characterizes typical characteristcs of spread and residual zones in terms of climate and environment, and explicitly links economic autonomy as a key condition in the genesis of residual zones. This is a clear and important point of articulation with the research on linguistic diversity that I have sketched above, in particular Nettle’s theory of ecological risk and economic autonomy or the lack thereof.

Spread and accretion zones thus can be thought to prototypes of very different linguistic landscapes, which are low in diversity on the one hand, and highly diverse on the other, against the backdrop of different economic and subsistence affordances for human societies. The crucial (prescient) contribution of these prototypes is that they are explicitly connected to different diachronic dynamics of language geography. A key relevant process is language expansion and diversification, the traditional forte of historical linguistics since the inception of the field. But what is of particular interest when it comes to explaining linguistic diversity, complementarily to the dynamics of spread zones, is the dynamics of residual zones – those parts of the world that major language spreads do not reach, or at least do not have an impact that would reduce their linguistic diversity significantly. Indeed, “often a residual zone will be located at the periphery of a spread zone” ( Nichols, 1992: 21) – consistent with the idea that the culmination point of language expansions is typically reached before these areas can be affected.

This is consistent with the now robust observation that language spread trajectories respond to environment ( Bentz *et al.*, 2018). For instance, the thrust of the Bantu expansion reflects ”a measureable preference for … familiar savannah Habitats“ ( Grollemund *et al.*, 2015: 13299) of the people driving it.

**Figure 4.**
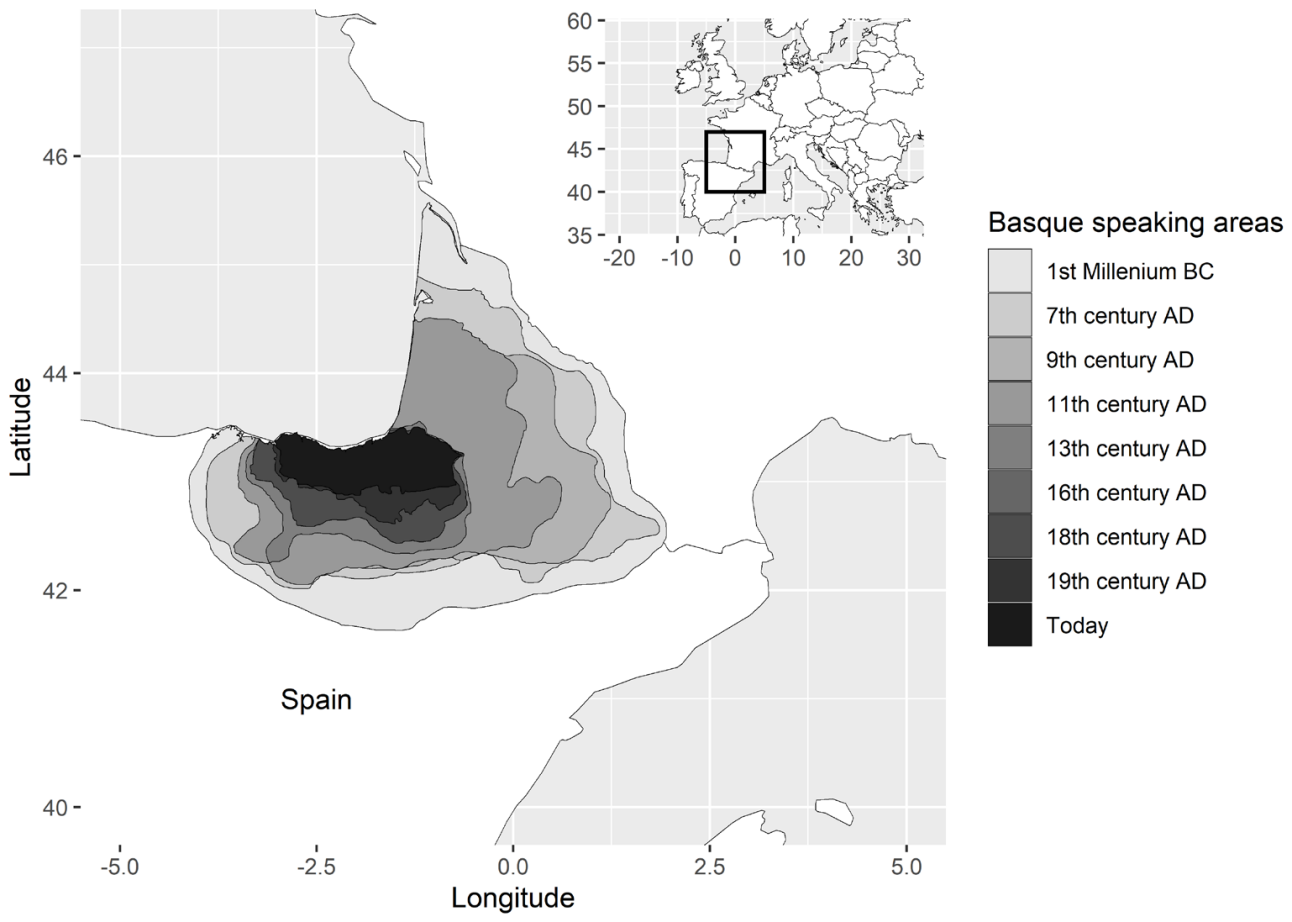
Historical changes in the geographical extent of the Basque language, from Urban (2021).

In Urban (2021), I have provided a perspective on such dynamics from the point of view of qualitative case studies on language isolates and how the ranges in which these languages are spoken have contracted in the course of attested history. Basque is a textbook example. Once spoken far into the Pyrenees and into the level Ebro valley of Northern Spain (in fact, Ebro goes back etymologically to a Basque word for ‘valley’), the domain of the language has gradually shrunk, starting in antiquity and continuing up to the present. Figure 4, from Urban (2021), illustrates the process. Why is Basque spoken today in exactly that part of its former range in which we find it rather than in another? Trask (1997) explains that ”the mountainous Basque terrain, with little agricultural land, no cities, few obvious resources, and harbours that faced uselessly (from the Roman point of view) onto the Atlantic, was simply too insignificant to be worth the trouble of colonization. And the same lack of Roman interest is very largely what guaranteed the unique survival of the Basque language” Needless to say, this also means that the expansion of Latin came to a halt, or was mitigated, before reaching the Basque country. In Urban (2021), I discuss also the historical language geography of Burushaski, which is largely parallel to that of Basque, but adds a vertical dimension to the relevant geographical processes (see also Nichols, 2013). Here I would like to point out that the case of Basque is just one example of a broader patterns of neolithic Europe, where languages that likely predate the Indo-European spread are conspiciously found in peripheral regions like Basque, or on islands, in other words, at the geographical margins of Europe We can adduce similar dynamics to explain linguistic distributions and the emergence of residual zones for which we cannot rely on historical evidence. In western Mexico and Mesoamerica, language isolates and small language families are found at the edges of major agricultural spreads, strongly suggesting a dynamics in which former, pre-spread language distributions were reduced to geographically and ecnonomically marginal regions. Similar processes are not restricted to the deep past. They can be observed about 1500 years later in the context of colonial regimes. For instance, on the Pacific coast and in the Andean highlands of South America, Spanish colonial administrators removed the Indigenous people from the agriculturally most productive lands and resettled them to less fertile regions and into mission towns, and they also show themselves in the context of government-backed settler colonialism of the kind that drove the US westward expansion. Neither are such processes restricted to demographic changes introduced by agriculturalist, state-level imperial societies. Evans and McConvell (1998) provide a model for explaining a significant language spread in hunter-gatherer contexts and the associated social and linguistic context, informed by deep-rooted Australian cultural practices.

More generally, the advantage of this process-based, range reduction oriented approach to studying linguistic diversity is that their net results are compatible with, and fact at least in certain cases predicted by, qualitative local language dynamics and ecologies. Nichols (2013) describes the traditional language dynamics of the Caucasus as one of asymmetric and gendered multilingualism which is embedded and dynamized by the traditional subsistence patterns in this mountain areas. Languages in the highland villages are typically community-based and the vehicles of communication for inward-facing “societies of intimates” ( Thurston, 1989). They are not or only very rarely learned by outsiders. The men of these communities, however, spend time in the lowlands to visit markets, and often spend the whole winter months in the lowlands, where herds would still find pasture. As such they are under pressure to learn languages of the lowlands, but lowlanders are under no pressure to learn highland languages. As a result, the general language dynamics of the Caucasus is one in which languages would constantly encroach the territory in an uphill direction, building up additional diversity without ousting that of the highlands that already exists. As a result, the oldest layers of the diverse linguistic landscape of the Caucasus would be found at the highest altitudes, according to this model which. Over the *longue durée* should accrete linguistic diversity both on the levels of language richness and phylogenetic diversity.

Finally, there is the question of the curious congruence between linguistic and biological diversity. While comparisons between language diversity and species diversity have frequently been made, this has, to the best of my knowledge, only concerned the static situation at present, just like investigations of linguistic diversity have mainly been static (though see Gavin *et al.*, 2013; Gavin *et al.*, 2017; Pacheco Coelho *et al.*, 2019). However, there is a point of articulation between both when conceived of in diachronic terms as I have sketched above. This point of articulation is the specific way how the geographical ranges of species and languages shrink and contract as they are pushed out of their former ranges, for instance by invasive species or anthropogenic factors in the case of species, and, in the case of languages, in case of expansion of a language or a language family that comes to be spoken in the regions that were formerly its domain and slowly or more quickly replaces them. This can happen in a variety of ways, including “demic diffusion” scenarios where indeed language spread is linked to actual migration of people, or through “cultural” mechanisms involving language shift.

Traditionally, biologists have thought that when the geographical range of a species contracts, this would likely begin in the peripheries of the region, which typically offer only less-than-optimal habitats and where the density of populations is more uneven and less dense. Hence, in peripheral regions individuals would be more vulnerable to disruptive factors, while core populations would be less so and therefore persist longer. However, Channell and Lomolino (2000) have shown through a study of how the geographical ranges of 245 species have contracted before they have become extinct or threatened by extinction due to habitat loss that the locales where the species survives the longest typically is situated exactly at the periphery of the larger, original range. For instance, the Tasmanian tiger ( *Thylacinus cynocephalus*) originally occurred throughout New Guinea and Australia, and received its name from its last refugium, the island of Tasmania at the southwesternmost periphery of the original range. The characteristics of these refugia as described by Channell and Lomolino (2000) are “those along the edge of the range, on an isolated and undisturbed island, or at high elevations”, a type of location that we are familiar by now – from language dynamics.

## Conclusion

Here, I have presented an overview on the puzzle of global language diversity, which, highly unevenly distributed across different regions of the world, is integrated with and sustained by a wide range of societies and their respective views on language, language diversity and what role they should play. I have presented a model for understanding language diversity that, in contrast to most extant work, is based on qualitiative case studies of how the range of languages contract in the wake of language expansion and what the characteristics of the places in which they survive longest are. This makes references to environmental, including climatic and geographic variables, that promote or inhibit the social, cultural, political, and economic dynamics that are associated with language spread and thus at the same time incorporates human agency. Furthermore, the model takes serious the fact that languages are cultural, not biological products, and does not require a brute evolutionary view on cultural and linguistic diversity, but still opens a perspective on the dynamics linguistic diversity that can be related meaningfully to the patently similar dynamics of biological diversity.

I acknowledge that there is a lot that is not yet understood, and that loose threads remain.

One point that I wish to highlight here is the general applicability of models for explaining language diversity based on dynamics of language range reduction and the accretion of linguistic diversity in residual zones. Here, I have suggested that such high-diversity zones arise because large-scale language expansion processes culminate before they are reached, and that they do so because of less favorable environments for speakers of spreading languages. However, accretion zones are also found in California, with a climate that provides suitable conditions for a reliable food and subsistence base year round – this is consistent with Nettle’s ecological risk hypothesis, but not necessarily the range reduction-based dynamic model that I have sketched here. Eventually, it may be the case that we must reckon with non-stationary effects of different environments on language diversity levels ( Pacheco Coelho *et al.*, 2019) and the way they shape the societies and their ideologies that sustain highly diverse linguistic landscapes. In other words, we might have to distinguish several types of residual zones (as is now done for spread zones: Nichols, 2015), created by different diachronic cultural and linguistic dynamics. This would also be consistent with the “non-stationary” nature of language ideologies, which express different attitudes towards multilingualism and which sustain different roles of language, language variation, and linguistic differences in constituting ethnic or social identities.

## Ethics and consent

Ethical approval and consent were not required.

## Data Availability

No data are associated with this article.
