# Global language geography and language history: challenges and opportunities

**DOI:** 10.12688/openreseurope.18421.3

**Published:** 2025-11-17

**Authors:** Matthias Urban

**Affiliations:** 1 Dynamique Du Langage, Centre Nacional de la Recherche Scientifique, Lyon, France; 2 Universite de Lyon, Lyon, France

**Keywords:** Language geography, language dispersal, linguistic diversity, typology, historical linguistics

## Abstract

While it has almost become a truism of comparative linguistics that linguistic diversity is unevently distributed across the globe, the underlying processes are poorly understood up to the present day. Linguists are thus in the embarassing situation that they do not understand significant regularities in the way the objects of their study –languages– pattern. In this essay, I explore three interrelated strands of thought to create a perspective on the question that is different from those explored so far: first, I suggest that instead of looking at present-day levels of diversity statically, we should take an approach that looks into how these distributions were generated. Related to this point and in contradistinction to extant work, second, I advocate an inductive approach which includes qualitative case studies that inform theory-building and allow empirical judgments on the propensity of certain environments to foster the emergence of certain linguistic landscapes. Third, I ponder that, in contrast to the traditional focus of historical linguistics on language diversification and expansion, understanding how the ranges of languages are reduced might be the key missing piece of evidence in a global theory of linguistic diversity and its genesis. This new perspective is also able to address the striking correlation between linguistic and biological diversity, which suggests that the processes that created and maintain both are, on some level, qualitatively similar.

## Plain language summary

This essay reflects on the distribution of linguistic diversity across the globe, which at present cannot be explained satisfactorily. I suggest that to understand this distribution we need to look at actual, concrete processes that have generated the ranges of individual languages, and that we can extrapolate from these to understand patterns in linguistic diversity at large.

## Introduction: The puzzle of linguistic diversity

During the 19th and 20th century, our knowledge concerning the diversity of human languages has increased massively. We know now that there are many more distinct languages spoken around the world than what has generally been thought possible some centuries ago, namely around 7000. We also know now that the way these languages function is massively more diverse than has been thought possible as well ( Evans & Levinson, 2009).

“Linguistic diversity” can mean several related things that must be distinguished carefully. First, the term can refer to the sheer number of languages relative to the size of a study area, e.g. a country (this is sometimes called “language richness”). Second, linguistic diversity” can reference the number of different language *families* relative to the size of a study area (this is sometimes referred to as “phylogenetic diversity”). These two parameters (which can be derived either by sheer counting or the computation of diversity indexes adapted from ecology, Peukert, 2017) correlate often, but not always. In Africa, for instance, the sub-Saharan belt hosts hundreds of clearly distinct languages, but many belong to the so-called Bantu branch of the Niger-Congo family that began to diversify several thousand years ago. Orthogonally to these two dimensions, finally, the languages in a given study area may also be very similar or very diverse in how they sound and how their grammars work (this parameter is sometimes also called “structural diversity”, Nettle, 1998).

The three parameters–language richness, phylogenetic diversity, typological diversity–define very distinct linguistic landscapes in different parts of the world.

In Vanuatu, for example, the approximately 9,400 inhabitants of the 50 villages on Banks and Torres Islands recognize 17 distinct languages. This is very high language richness relative to the number of inhabitants and surface area. However, these form part of the Oceanic subgroup of the vast Austronesian language family, and within that group, are more or less closely related to one another.

**Table:** 

Hiw	sisə	tati	jɵjmə ^g^ʟen	wu ^g^ʟɔɣ	kʷe	i	nə	məŋa	ta
Lo-Toga	nihə	tat	lolmərɛn	ʉrβɛ	kʷɛ	e	nə	βəɣəβaɣə	məʈə
Lehali	kɛj	tɛtnɛ	ɣlal	ɣalsɛ	kʷɒ		n-	βap	munɣɛn
Löyöp	k͡iɛj	tɛ	ɣilal	ʧøjmat	ʧɛk͡pʷɛ		n-	βaβap	ŋ͡mʷɔn͡iɛn
Volow	^ŋ^gɪj	ɛt	ɪɣlal	ɣalsi	tɛ ^ŋ^g͡bʷɛ		n-	ɣatɣat	njɔnɣɪn
Mwotlap	kɪj	ɛt	ɪɣlal	ɣalsi	k͡pʷɛtɛ		nɔ-	hɔhɔlɛ	nɔnɔnɣɪn
Lemerig	tær	ɪ	ɣɒlɒl	ʔørmaʔ	ʔæ.kiʔis		n-	tɛktɛk	mʊɣʊt
Vera’a	ⁿdir	ɪʔ	lamai	ɛntɛɣ	ʔɪn		ɪn	tɪktɪk	muⁿdɪ
Vurës	nɪr	ɣɪtɪ-	ɣilal	warɛɣ	tɛn		ɔ	k͡pʷak͡pʷ	namøɣynɪn
Mwesen	nɪr	ɛtɛ	lɪlɪ	maŋtɛ	βɪs		ɔ	ɣatlɛ	mɔɣɔnin
Mota	nira	ɣate	ɣlala	mantaɣ	tk͡pʷe		o	βaβae	naŋ͡mʷunina
Nume	nir	βitis	ɣil	liŋliŋi	mi		u	luwluw	namɣin
Dorig	nɪr	sɔwsɛ	βrɪɣɪl	taβul	tɛ		na	lŋa-	ɣɪn
Koro	nɪr	tɪ	rɔŋ	taβul	wʊs.mɛlɛ		ɔ	βalβalaw	namɪɣɪn
Olrat	nɪj	tɪ	rɔŋ	βɪlɪː	wʊs.mɛlɛ			ususraː	mʊʧ
Lakon	ɣɪː	atɪ	rɔŋ	kɛrɛ	aβʊh.malɛ			ɛlŋa-	nɣɪʧ
Mwerlap	kɛr	ti	βalɣɛ͡ar	mɪnmɪn	tɪkʷɪtɛ͡a		nɞ-	liŋɪ-	ɣɛ͡an
	3 pl	not.yet_1_	know	properly	not.yet_2_	[ obl]	art	speech	poss:1 incl.pl

Above is the sentence *They don’t know our language very well yet* in each of these 17 languages, collected by Alexandre François (2011). In spite of lexical differences, the structure of the sentence in each of the 17 languages is remarkably similar, so that one can sometimes translate word by word from either one of the Banks and Torres Islands languages to any other because their grammars work in similar ways. In sum, with 17 languages, language richness is very high on these islands; phylogenetic diversity is very low as they are all related to one another; and structural diversity likewise is low.

Northern Eurasian languages score relatively low on all three counts: there are relatively few different languages; these belong to a small number of distinct language families; and most, in particular those resulting from recent language spreads, are quite similar in how they sound and how their grammars work.

These are some examples of the myriad different ways in which the three parameters of diversity can combine.

In this essay, I will mainly be using the term “linguistic diversity” in the sense of “language richness”. However, the research program I sketch makes reference also to different levels of typological diversity in different parts of the world in ways that follow from the same general underlying reasoning.

Visualizing linguistic diversity in an accessible way is not easy and requires a number of decisions and qualifications. While there are parts of the world where relatively clear language boundaries exist, this is not the case everywhere. Languages may overlap in geographical space in complex ways, either because speakers of more than one language live next to each other in the same communities, or because languages “live” next to each other in the minds of bi- or multilingual speakers (societal vs. individual bi- or multilingualism, Appel & Muysken, 2005), or both. Any representation of language on maps is inadequate to capture these distributions in their full complexity ( Luebbering, 2013; Urcioli, 1995), and for most of the world’s languages, such detailed information is simply unavailable anyhow.

**Figure 1.**
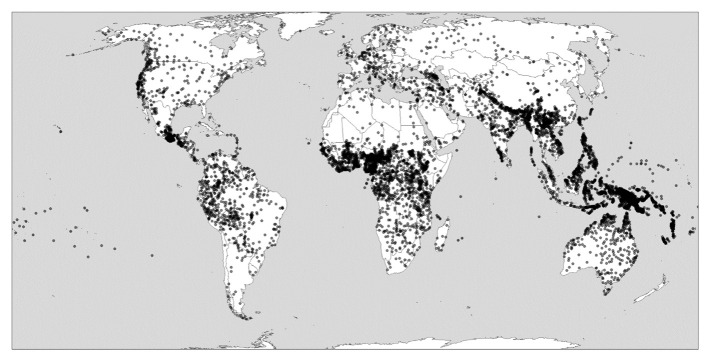
Global linguistic diversity (language richness). Created using the R package glottospace ( Norder *et al.*, 2022).

Figure 1 nevertheless attempts to show globally uneven levels of language richness. It represents languages as dots in the absence of widely available polygon data that could represent the ranges in which languages are spoken more realistically, let alone more detailed information on the distribution of languages in social space. ^1^ Coordinates come from Glottolog ( Hammarström *et al.*, 2024), the leading catalogue of the world’s languages via the glottospace R package ( Norder *et al.,* 2022). In the case of small-scale languages, dots typically represent the center of the area where the language is spoken; in other cases, historical or demographic information inform decisions (as when Russian is plotted at the coordinates of Moscow).

**Figure 2.**
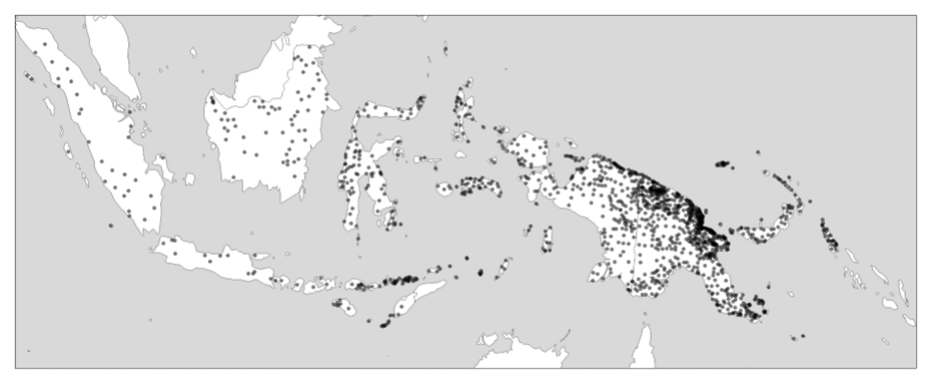
Linguistic diversity (language richness) in Indonesia and Papua New Guinea. Created using the R package glottospace ( Norder *et al.*, 2022).

Asymmetric levels of language richness are not only observed within continent or island-sized areas such as those I have just mentioned, but scale down to variation *within* such areas: In New Guinea, it is the coastal areas in the north that host disproportionally much of the island’s language richness, while the New Guinea highlands, which traverse the island longitudinally, are lower in diversity both measured in terms of individual languages and in terms of different families. Figure 2 gives an impression of this.

**Figure 3.**
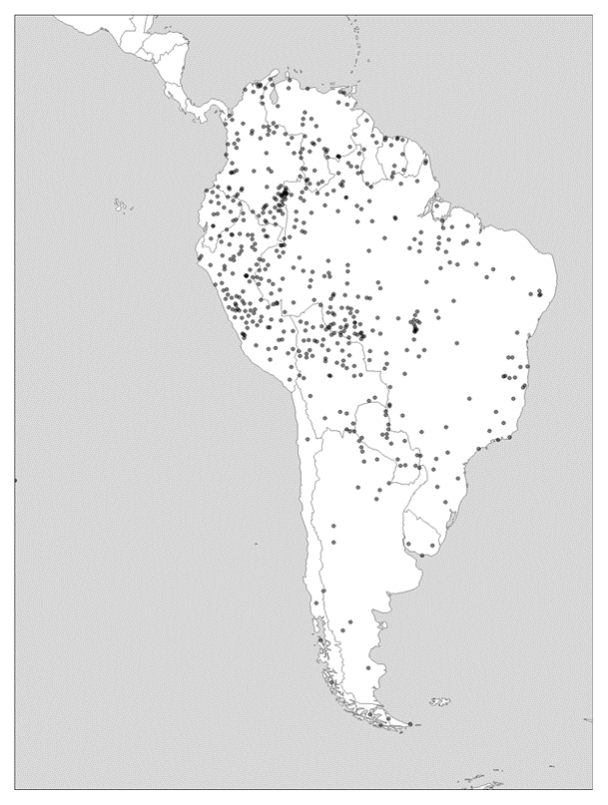
Linguistic diversity (language richness) in South America. Created using the R package glottospace ( Norder *et al.*, 2022).

As can be observed in Figure 3, in South America, diversity levels are high throughout greater Amazonia, but particularly pronounced on the eastern margins. In the Andes themselves, and as one moves southward to Patagonia and Tierra del Fuego, language richness and phylogenetic diversity becomes notably lower. In North America, it is only California that boasted a hyperdiverse mosaic of languages or language families, whereas to the east of the Rockies, diversity is measurably lower.

It is such observations of nested diversity clines that suggest that it is no mere coincidence of history that New Guinea, Amazonia, and California are hyperdiverse, whereas Greenland, Patagonia, and eastern north America are not to the same extent. However, up to the present day, the reasons for regionally uneven diversity levels are not understood.

As the differential patterning of linguistic diversity has come to the attention of linguists and scholars in other disciplines in the early 21st century, a striking congruence with biological diversity was observed ( Gorenflo *et al.*, 2012; Maffi, 2005; Moore *et al.*, 2002). Also this relationship is not only observed globally, but on intra-continental scales, too (though at some levels of resolution, it breaks down: Manne, 2002). For instance, in South America, the Amazonian fringe and the cloud forest ecotone at the intersection of Andes and eastern lowlands are not only hotspots of linguistic, but also biological diversity.

Languages are cultural products, shaped by the communicative behaviour of their speakers ( Du Bois, 1987) and the social ecologies they are embedded in ( Pakendorf *et al.*, 2021; see more extended discussion in the following sections). They are *not* species. Drawing an analogy between biological evolution and “cultural evolution” is as tempting as potentially dangerous and simplifying, especially as we have a lot of qualitative knowledge on the maintenance of linguistic diversity and their social ecologies that are specifically human (again, more extended discussion is in the following sections). Still, undeniably, languages cover geographical space in ways that are strikingly similar to the ways in which biological species do.

Like Hua *et al.* (2019), I tend to view this association as epiphenomenal (linguistic and biological diversity co-vary with climate and/or environment, which shapes the distribution of both) rather than causal (humans diversify culturally in areas with high biodiversity more readily because of the richness of available resources).

The latter scenario would be consistent with a prominent hypothesis that has been suggested to explain the uneven linguistic diversity of the world. In the next section, I turn to current perspectives on linguistic diversity, including this hypothesis of “ecological risk” ( Nettle, 1999). I also discuss some of the issues I see with extant work, and questions that still remain open, either because studies so far have yielded contradictory answers or because they have not been a focus of attention.

## Current perspectives (and their problems)

### Explaining global linguistic diversity: Emergence and maturation of a research field

There are minimally three dimensions to the problem of explaining global linguistic diversity: first, there is the question which types of environments (if any are identifiable) are conducive to high linguistic diversity and which mitigate its development; second, there is the question if the congruence between cultural and biological diversity is a coincidence, and, if not, how it should be explained; and third, especially because languages are not species, there is the question how such factors, if they could be identified, link up with actual human linguistic and non-linguistic behavior.

In the last 25 years or so, a number of studies have appeared that address the question of linguistic diversity and its drivers. However, they have mainly focussed on the first dimension, while the actual behaviour of people that, in the long term, could lead to these distributions has received much less attention. I will return to this point later.

Early studies ( Cashdan, 2001; Collard & Foley, 2002; Hillebrand, 2004; Mace & Pagel, 1995) mainly noted a latitudinal gradient to global levels of linguistic diversity (see also Gavin & Stepp, 2014).

Nettle ( 1996; 1998) was an influential pioneer in singling out a possible explanation, and his “ecological risk” hypothesis is still viable today: Nettle argued that linguistic diversity in the sense of language richness correlates not just with latitude, but more directly with the length of the Mean Growing Season, i.e. the number of months per year that allow, given local conditions like temperature, rainfall, etc., vegetation to grow. The length of the Mean Growing Season, in turn, is taken by Nettle as a proxy for “ecological risk”: Where it is long, ecological risk is low, and societies are hypothesized to be self-sufficient: the environment allows enough resources for most parts of the year to ensure reliable subsistence. Such conditions are said to foster the development of many small, self-reliant speech communities that do not need to maintain outside ties to safeguard against supply shortages. Where climatic conditions are less stable, or where resources are scarcer due to a shorter Mean Growing Season, people must increasingly derisk their subsistence base. This involves the exploitation of resources on larger tracts of land, and/or the maintenance of stronger inter-community ties and larger networks – hence, so the hypothesis, fewer speech communities spread out over wider ranges ^2^.

In the meantime, the size of the data analyzed, the number of possible environmental variables considered, and the methodological sophistication of relevant work has grown enormously. Spatial autocorrelation, a typical phenomenon in spatially structured data, has been shown to confound results in research on the environmental and social conditioning factors of linguistic diversity ( Cardillo *et al.*, 2015). It has also been noted that the effect of surveyed climatic variables may be non-stationary and interact in locally specific ways in generating linguistic diversity or inhibiting it ( Pacheco Coelho *et al.*, 2019). As a whole, research on the drivers of linguistic diversity, reassuringly, shows the typical signs of maturation of a field of scientific investigation.

At the same time, however, the results of relevant studies that have appeared after Nettle differ widely. We have now a whole series of articles, often in high-standing journals and using sophisticated methodology, that share the general concern of identifying ecological drivers of linguistic diversity, and that assess a wider range of environmental parameters rather than just Mean Growing Season as a proxy for ecological risk. Axelsen and Manrubia (2014) identify the presence of rivers and terrain roughness (technically, “rugosity”) as factors that are conducive to the rise of linguistically diverse landscapes – but earlier Currie and Mace (2009) only found a weak effect of rugosity (which they measured differently, however). While Sutherland (2003) found no evidence for Nettle's ecological risk hypothesis, Hua *et al.* (2019) found the effect of geology-related variables like rugosity to be relatively negligible compared to climate and year round productivity, consistent with an account like that of Nettle. Derungs *et al.*’s (2018) results are likewise consistent with the “ecological risk” hypothesis, but they conclude that climate –latitude, precipiation, and temperature – is more relevant for linguistic diversity of food-producing (agriculturalist) societies than for hunter-gatherers, which would mean that “ecological risk” should affect different types of people in different ways.

### Language ideologies, diversification trajectories, and macro-distributions of linguistic diversity

While the question which environmental or climatic conditions foster linguistic diversity thus remains open for the time being, a broader question is how *any* climatic and environmental factors could lead to actual linguistic and non-linguistic behavior of people that, in turn, sets into motion processes that would generate observed patterns of diversity. As sketched above, an answer to this question is encapsulated in the “ecological risk” hypothesis. However, as far as I am aware, that ecological risk actualizes linguistic behavior with repercussions on language richness, or even phylogenetic and structural diversity, has so far never been shown through detailed qualitative case studies, but only stipulated by hypothesis.

Where case studies on the ecology of linguistic diversity in the tradition of the ethnography of communication ( Hymes, 1964) exist, it often turns out that it is language ideologies of one kind or another that sustain diversity in arrangements of so-called small scale multilingualism ( Pakendorf *et al.*, 2021).

One well-described case again comes from Vanuatu ( François (2012): forms of speech, whether linguists would classify them as dialects or languages, are considered to be locally anchored to communities in individual villages, as are other cultural activities such as ways of preparing food, singing etc. This link between place and language is valued and actively maintained and fosters the development of locally specific speech forms, including details of pronunciation and vocabulary that come to be viewed as typical of different places – and these are precisely those highly salient features of speech that people are likely to note and associate with different languages. At the same time, there is “egalitarian multilingualism” ( Haudricourt, 1961; Vaughan & Singer, 2018): no language is considered per se more valuable than any other (even though, in practice, some languages are particularly important because they have disproportionally high speaker numbers). People do not mind learning the languages of others, also because marriages are typically exogamous so that multilingual households are common. Such kinship ties are complemented by commercial ones, and together they lead to dense networks of social and commercial interaction. This, of course, is fertile ground for convergence effects such as the ones reflected in the paralellism in grammar between Vanuatu languages. In sum, the particular linguistic ecology of Northern Vanuatu fosters both differentiation and fragmentation with high language richness and low structural diversity: that is, exactly the configuration of linguistic diversity that can be observed.

On the other hand, other qualitative, “thick” ( Geertz, 1973) descriptions of language ecologies suggest that notions like “group boundary formation” ( Gavin *et al.*, 2013), i.e. the essentializing use of a form of speech to identify one’s “ethnic group”, set it off against other such groups, and thus yield “ethnolinguistic groups”, are far from universal. Nettle himself, who relies on the “ethnolinguistic group” as a unit of analysis, quotes Brooks (1993: 27) to the effect that in West Africa, “individuals and families change their language and modify their social and cultural practices in ways that are often perplexing to outsiders.” In other words, language is not always firmly linked to particular individuals that together would make up an “ethnolinguistic group”, but language use may be dependent on social context, roles, and local language ideologies. An influential analysis of such configurations in the Balkans is in Irvine and Gal (2000); for the ancient and present-day Central Andes in Babel (2018) and Urban ( 2018; To appear); and for Upland Southeast Asia and the Himalayas in Scott (2009) and Shneiderman (2010). As Evans (2018: 13) aptly puts it, “the focus on languages diversity as something manifested by discrete, internally coherent entities can remove the very types of evidence we need to tackle the diversification problem.”

In sum, the qualitative literature suggests that the recruitment of languages as markers of local identity and groups, while existing also in some “traditional” contexts, is far from universal. General, one-size-fits-all explanations for the genesis of linguistic diversity are thus problematic.

More specifically, in direct contradiction to the idea that ecological risk fosters a smaller number of languages with wider ranges, in traditional settings, it is precisely linguistic diversity which, sustained in multilingual landscapes, can be observed as a strategy "that maximizes alliances and protective networks through different languages“ ( Lüpke, 2016: 53).

At present, thus, research on global linguistic diversity is in a remarkably open situation. The questions are on the table, but I think it is fair to say that answers that are robust to different analytic approaches and datasets and that are consistent with actually observed linguistic and non-linguistic behaviour have not crystallized yet. The impasse pertains most pressingly to the first two dimensions of linguistic diversity that I have sketched in the introduction. Astonishingly, linguists do not understand the ways in which their objects of study –languages– are distributed on the largest scales, and especially why they are distributed this way. This is in contrast to the micro-levels of variation. Dialectologists, since the 19th century, have developed methods to describe and (at least to some extent) explain how features of pronunciation, grammar, and words change in geographical space, and the entire discipline of variationist sociolinguistics explores how language varies in social space. It is also in contrast to what we, as linguists, know on the distribution of features across whole languages and large regions (a field of study often called “areal typology”, in which clines and very large skewings, not dissimilar to those concerning linguistic diversity, have been observed up to continental scales – Bickel, 2020; Dryer, 1989; Güldemann & Hammarström, 2020; Nichols, 1992; Urban *et al.*, 2019), and what we are beginning to learn on how the social ecologies in which languages are learned and spoken influence their structure and complexity (e.g. Lupyan & Dale, 2010; Trudgill, 2011). The situation is also relevant to the human sciences at large as the processes by which human societies produce those key cultural products which they have used to define themselves since antiquity –languages–- remains in the dark.

## Dynamizing linguistic diversity

### A research program

As the main contribution of this essay, in this section I explore lines of reasoning that might lead to perspectives on linguistic diversity and its origins that is able to bring together the abovementioned perspectives more satisfactorily. Since this is an essay, my discussion is mainly programmatic; whether the picture I will try to get into focus has merit is an open question.

I want to make three interrelated points: first, I suggest that instead of attempting to find parameters of variation (climatic, environmental, political, etc.) between regions with differential levels of linguistic diversity, a process-based approach that looks into how these distributions were generated can furnish perspectives that would be otherwise missed. In this vein, I suggest a way to dynamize the question of linguistic diversity and its drivers in a way that references the observed congruence between linguistic and biological diversity. ^3^ Second, I ponder that, in contrast to the traditional focus of historical linguistics on language diversification and expansion, understanding how the ranges of languages contract might be the key missing piece of evidence in a global theory of linguistic diversity and its genesis. Related to this point and in contradistinction to extant work, third, I advocate an inductive approach that departs from qualitative case studies which both inform theory-building and allow empirical judgments on the propensity of certain environments to foster the emerge of certain linguistic landscapes.

### Spread zones, residual zones, and the dynamics of linguistic diversity

The starting point for developing my argument is the well-known distinction between spread and accretion (or residual) zones. Nichols ( 1992; 1997) distinguishes these as prototypical and contrasting types of language distributions in geographical space, and sketches their underlying dynamics. The dichotomy references the uneven distribution of linguistic diversity and at the same time contains elements of a theory to account for these dynamically.

As far as language geography is concerned, spread zones are dominated by relatively few language families (i.e. have low phylogenetic diversity), and at times even just contain few individual languages (i.e. have low language richness). These distributions are shaped by frequent and long-distance language expansions, which tend to completely or almost completely obliterate preexisting languages. These may include the languages that spread via earlier episodes of such expansions (“spread-over-spread” dynamics). As is expected in situations of rapid and long-range language spreads, the spreading languages are quite similar to one another as a result of the little time available for diversification through language change (i.e. low typological diversity). Thus, in spread zones, which languages and language families dominate the linguistic landscape can change drastically and quickly, but net linguistic diversity does not: it remains low on all three counts.

Accretion zones, in the definition of Nichols (1992: 14–15), have the following characteristics: they contain old families (i.e. ones that are deeply diversified internally – this does not necessarily mean that these families must contain many individual languages, but that the languages belonging to its different branches are not closely related, which indicates a long time of internal differentiation). “Old families” in the relevant sense should be taken to include language isolates, which are the sole representative of lineages that are so old that relatives cannot be detected anymore. No major language expansions originate from accretion zones, but they may attract instrusive languages and thus serve as a linguistic “refugium of sorts” in Nichols’s words. The arrival of these languages, however, does not lead to significant levelling of preexisting linguistic diversity in the accretion zone; rather, in addition to processes of diversification through language change that take place relatively undisturbedly, they contribute further to a accretion zone’s linguistic diversity.

Nichols (1992) also characterizes typical characteristics of spread and accretion zones in terms of climate and environment, and explicitly mentions economic autonomy as a key condition for the genesis of accretion zones. This is a clear and important point of articulation with the research on linguistic diversity that I have sketched above, in particular Nettle’s theory of ecological risk and economic autonomy or the lack thereof, but at the same time opening an access point to a dynamic, process-based understanding of linguistic diversity.

Spread and accretion zones can thus be thought of as prototypes of very different linguistic landscapes that form against the backdrop of different economic and subsistence affordances for human societies. The crucial (prescient) contribution of these prototypes is that they are explicitly connected to different diachronic dynamics of language geography.

A key relevant process is language expansion and diversification, the traditional forte of historical linguistics since the inception of the field. But what is of particular interest when it comes to explaining linguistic diversity, complementarily to the dynamics of spread zones, is the dynamics of accretion zones – those parts of the world that major language spreads do not reach, or where they at least do not have an impact that would reduce linguistic diversity significantly. Indeed, “often a residual zone will be located at the periphery of a spread zone” ( Nichols, 1992: 21) – consistent with the idea that the culmination point of language expansions is typically reached before these areas are affected. This is in line with the now robust observation that language spread trajectories respond to the environment ( Bentz *et al.*, 2018). For instance, the thrust of the Bantu expansion reflects ”a measureable preference for … familiar savannah habitats“ ( Grollemund *et al.*, 2015: 13299) of the people driving it.

### How language ranges contract

**Figure 4.**
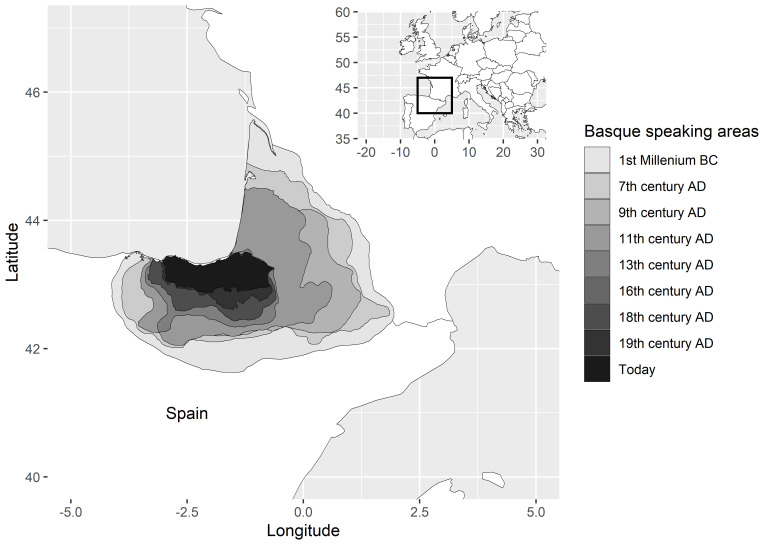
Historical changes in the geographical extent of the Basque language, from Urban (2021).

In Urban (2021), I provide a perspective on such dynamics through qualitative case studies on language isolates and how the ranges in which these languages are spoken have contracted in the course of attested history. Basque is a textbook example. Once spoken far into the Pyrenees and into the Ebro valley of Northern Spain (in fact, Ebro goes back etymologically to a Basque word for ‘valley’), the domain of the language has gradually shrunk, starting in antiquity and continuing up to the present. Figure 4, from Urban (2021), illustrates the process. Why is Basque spoken today in exactly that part of its former range in which we find it rather than in another? Trask (1997) explains that ”the mountainous Basque terrain, with little agricultural land, no cities, few obvious resources, and harbours that faced uselessly (from the Roman point of view) onto the Atlantic, was simply too insignificant to be worth the trouble of colonization. And the same lack of Roman interest is very largely what guaranteed the unique survival of the Basque language”. Needless to say, this also means that the expansion of Latin came to a halt, or was mitigated, before reaching the Basque country. ^4^ Basque is just one example of a broader patterns in neolithic Europe, where languages that likely predate the Indo-European spread are conspiciously found in peripheral regions like Basque, or on islands, in other words, at the geographical margins of Europe.

One can hypothesize similar dynamics to explain linguistic distributions and the emergence of accretion zones for which we cannot rely on historical evidence. In western Mexico and Mesoamerica, language isolates and small language families are found at the edges of major agricultural spreads, suggesting a dynamics in which former, pre-spread language distributions were reduced to geographically and economically marginal regions. Similar processes are not restricted to the deep past. They can be observed about 1500 years later in the context of colonial regimes. For instance, on the Pacific coast and in the Andean highlands of South America, Spanish colonial administrators removed Indigenous people from the agriculturally most productive lands and resettled them to less fertile regions and into mission towns. They also show themselves in the context of government-backed settler colonialism of the kind that drove the US westward expansion. Neither are such processes restricted to demographic changes introduced by agriculturalist, state-level imperial societies. Evans and McConvell (1998) provide a model for explaining a significant language spread in hunter-gatherer contexts, informed by deep-rooted Australian cultural practices.

The advantage of this process-based, range reduction oriented approach to studying linguistic diversity is that its net results are compatible with, and in certain cases predicted by, qualitative local language dynamics and ecologies. Vanuatu as described by François (2012) is one such example. Another concerns the Caucasus as a prototypical accretion zone. Nichols (2013) describes the traditional language dynamics of the Caucasus as one of asymmetric and gendered multilingualism which is embedded in and dynamized by the traditional subsistence patterns in this mountain area. Languages in the highland villages are typically community-based and the vehicles of communication for inward-facing “societies of intimates” ( Thurston, 1989). They are not or only very rarely learned by outsiders. The men of these communities, however, spend time in the lowlands to visit markets, and often stay the whole winter months in the lowlands, where herds would still find pasture. As such they are under pressure to learn languages of the lowlands, but lowlanders are under no pressure to learn highland languages. Thus, the general language dynamics of the Caucasus is one in which languages would constantly encroach the territory in an uphill direction, building up additional diversity without ousting that which already exists. As a result, the oldest layers of the diverse linguistic landscape of the Caucasus would be found at the highest altitudes, according to this model. Over the *longue durée* the Caucasus should accrete linguistic diversity both on the levels of language richness and phylogenetic diversity.

### A new view on the relationship between linguistic and biological diversity

Finally, there is the question of the curious congruence between linguistic and biological diversity. The model also has advantages here. While comparisons between linguistic diversity and species diversity have frequently been made, this has, to the best of my knowledge, only concerned the static situation at present, just like investigations of linguistic diversity have mainly been static (though see Gavin *et al.*, 2013; Gavin *et al.*, 2017; Pacheco Coelho *et al.*, 2019). However, there is a point of articulation between both when conceived of in dynamic terms. This point of articulation is the specific way how the geographical ranges of species and languages shrink and contract as they are pushed out of their former ranges. This may happen by invasive species or anthropogenic factors in the case of species, and in the case of languages by the expansion of a language or a language family that comes to be spoken in regions that previously had had other languages (as we have seen for Basque).

Traditionally, biologists have thought that when the geographical range of a species contracts, this would likely begin in the peripheries of the region, which typically offer only less-than-optimal habitats and where the density of populations is less even and dense. Hence, in peripheral regions individuals would be more vulnerable to disruptive factors, while core populations would be less so and therefore persist longer. However, Channell and Lomolino (2000) have shown that the locales where species survive the longest typically are situated exactly at the periphery of the larger, original range. For instance, the Tasmanian tiger ( *Thylacinus cynocephalus*) originally occurred throughout New Guinea and Australia, and received its name from its last refugium, the island of Tasmania at the southeasternmost periphery of the original range. The characteristics of these refugia as described by Channell and Lomolino (2000) are “those along the edge of the range, on an isolated and undisturbed island, or at high elevations”, a type of location that we have encountered before – in language dynamics.

### From empirical case studies of range reduction to generalizations on linguistic diversity

In those regions of the world with long-standing Indigenous traditions of writing, we are in some cases lucky enough that we can trace changes in the distribution of languages in space through time over millenia. Basque is an example of such a fortunate case. In many parts of the world, however, the time depths for which we can trace language history is heavily limited by the inavailability of written sources from which we could infer older stages of languages and their distribution in space. In such regions, language distributions can be known only from the point of European colonization onward, and more detailed descriptions are in many cases only available significantly after that. Nevertheless, ancillary sources like the distributions of toponyms with characteristic elements that can be attributed to a certain language or language group, in some cases can give time-depth to language distributions in space also in the absence of direct historical documentation.

In most parts of the world, Indigenous languages are under pressure, and are increasingly replaced by the official languages of modern national states whose use promises possibilities of upward social mobility and economic opportunities. The result is that many minority languages are not used anymore across the whole range in which they are known to have once been spoken, but are increasingly restricted to certain parts of that original distribution, or have, as the end result, become dormant

While a major issue for the language sciences and the cultures that once sustained it, the pressure under which Indigenous languages find themselves affords a “geography-strong” ( Di Carlo & Pizziolo, 2012) historical linguistics the possibility to empirically compare distributions of languages in space at different points of time, like we can for Basque. And once these differences are studied across a number of individual cases, it becomes possible to ask broader questions: how does the landscape and climate of the places in which languages survive longest (or have survived longest before perhaps eventually becoming dormant) differ from the places in which they were once spoken? Is the terrain more rugose (hence less accessible), is it less productive for human subsistence, or, to make the link with extant theorizing, does it perhaps differ in measurable ways in Mean Growing Season because of differences in temperature or rainfall? These differences can be quantified precisely (in the simplest possible form by comparing summary statistics of polygons representing original and reduced distributions across languages), and in turn they allow us to characterize different types of environments in terms of their potential for preservation of languages and linguistic distributions that have been ousted elsewhere – very much like Nicholsian accretion zones, but derived in a radically empirical way.

And once this crucial extrapolation from known cases of language range contraction to generalized geographical-climatic profiles is made, a suite of questions relating to linguistic diversity can be asked and answered empirically: we can compare levels of language richness and phylogenetic diversity in areas like those to which, in known instances, languages have tended to get restricted to when coming under pressure from one or more expanding languages. We can also compare whether the typological profiles we find in them differ from surrounding areas, dovetailing with the idea that certain types of environments host typological profiles that may have once been more common generally but replaced in areas with more spread-potential by later language spread ( Efrat-Kowalsky *et al.*, 2023). In general, we can formulate a spatially explicit, process-based account of linguistic diversity, informed and dynamized by spatial processes of language expansion and retraction.

## Conclusion

Here, I have presented an overview on the puzzle of global linguistic diversity. Highly unevenly distributed across different regions of the world, linguistic diversity is integrated with and sustained by societies and their respective views on language, linguistic diversity, and what role it should play. I have presented a model for understanding linguistic diversity that, in contrast to most extant work, is based on qualitiative case studies of how the range of languages contract in the wake of language expansion. This model makes references to environmental variables that promote or inhibit the social, cultural, political, and economic dynamics that are associated with language spread and thus leaves room for human agency. Furthermore, the model takes serious the fact that languages are cultural, not biological products, and does not require a deterministic view on cultural and linguistic diversity. At the same time, it still opens a perspective on the dynamics of linguistic diversity that can be related meaningfully to the patently similar dynamics of biological diversity.

I acknowledge that there is a lot that is not yet understood, and that loose threads remain. One point that I wish to highlight here is the general applicability. I have suggested that high-diversity zones arise because large-scale language expansion processes culminate before they are reached, and that they do so because of less favorable environments for speakers of spreading languages. However, accretion zones are also found in California, with a climate that provides suitable conditions for a reliable food and subsistence base year round – this is consistent with Nettle’s ecological risk hypothesis, but not necessarily the range reduction-based dynamic model that I have sketched here. Eventually, it may be the case that we must reckon with non-stationary effects of different environments on linguistic diversity levels ( Pacheco Coelho *et al.*, 2019) and the way they shape ideologies that sustain highly diverse linguistic landscapes. In other words, we might have to distinguish several types of accretion zones (as is now done for spread zones: Nichols, 2015), created by different diachronic cultural and linguistic dynamics. This would also be consistent with the “non-stationary” nature of language ideologies, which express different attitudes towards multilingualism and which involve different roles of language, language variation, and linguistic differences in constituting ethnic or social identities.

## Ethics and consent

Ethical approval and consent were not required.

## Data Availability

No data are associated with this article.

## Footnotes

^1^ More technically, the dots represent “languoids” – a neologism introduced in Cysouw & Good (2013) for linguistic entities that may correspond to language families, languages, or dialects in traditional parlance. Boundaries between these widely familiar categories are not sharply defined – for instance, a set of dialects that are so divergent that speakers have difficulties understanding each other is not very different from a small family of closely related languages. The notion languoid allows reference to linguistic entities recognized in the literature without enforcing permanent decisions on their position on the dialect–language–language family continuum, and Glottolog allows for flexible “upgrading” or “downgrading” of the classification level as more information becomes available. The dots show linguistic entities with identifiers that are currently considered “language-level” in Glottolog ( Forkel & Hammarström, 2022), based on the absence of mutual intellegibility with any other language ( Hammarström *et al.*, 2024).

^2^ This should in principle be relevant both for agrictulturalist and more “traditional” lifestyles involving hunting and gathering, as ultimately plants form the basis for subsistence in both cases.

^3^ This parallels how language typology has been dynamized by not looking at static synchronic features of languages, but at the historical processes of language change that shape how languages function at present (e.g. Cristofaro, 2019; Greenberg, 1978).

^4^ In Urban (2021), I also discuss the historical language geography of Burushaski, which is largely parallel to that of Basque, but adds a vertical dimension to the relevant geographical processes (see also Nichols, 2013).
