# Supplementary material for: Global language geography and language history: challenges and opportunities
Source: Open Res Eur. 2025 Nov 17;4:213. Originally published 2024 Oct 7. [Version 3] doi: 10.12688/openreseurope.18421.3 (PMC11612551; doi:10.12688/openreseurope.18421.3)
Supplement: Peer review history

# Reviewer report: Reviewer response for version 3

**DOI:** 10.21956/openreseurope.21868.r64282

**Published:** 2025-12-29

**Authors:** Tuomas Väisänen

**Affiliations:** 1 Department of Geosciences and Geography, University of Helsinki, Helsinki, Uusimaa, Finland; 2 Helsinki Institute of Sustainability Science, University of Helsinki, Helsinki, Uusimaa, Finland; 3 Helsinki Institute of Urban Regional Studies, University of Helsinki, Helsinki, Uusimaa, Finland

**Statement**

 The manuscript has improved nicely since the latest revisions, and I have only one moderate/minor concern and several very minor concerns left. The moderate concern relates to the remaining ambiguity to what the proposed process-based and range-reduction-oriented approach actually is. To remedy this, I suggest adding an informative figure that would illustrate the approach and visually juxtapose it with previous approaches, to make it crystal-clear. Alternatively, I suggest adding a paragraph where this is clearly defined.

 The minor concerns relate to textual clarity. First, there are several one sentence paragraphs here and there, which I find hard to find the reason for, as they would easily fit into paragraphs above or below them. They break up the flow of the MS and do not seem to have a consistent theme or a stylistic purpose. Second there are also several instances of wordy language, such as "the ways in which" which could just be said as "how", and some sentences have numerous subordinated clauses that make it difficult to follow the author's train of thought. Similarly, some of the sentences are quite long and the MS would benefit from splitting them into separate sentences. Third, there are occasions in the MS where the author is referring to another part in the MS, but in a vague manner, such as "I will come back to this point later", I suggest adding clear references to sections to such occasions. Related to this, the author often uses words like "this", "those", and "these" to reference something stated earlier, instead of writing out what the author is actually talking about, which makes understanding some statements rather difficult. Finally, and as mentioned in my previous review, there also are a few instances of the author referring to a theory or a statement made by a central author (like Nettle or Nichols), without providing a reference to go with it.

 Thus, I recommend minor revisions. I have provided detailed comments to highlight most of these concerns I mentioned above, but ask the author to check the MS throughout. However, I look forward to seeing the revised MS, which I believe will be in a very acceptable condition by next round.

**Detailed comments**

**Puzzle of linguistic diversity**

- Can you integrate the sentence "These are some examples of the myriad different ways in which the three parameters of diversity can combine." into the text above it, as it now stands alone as a one sentence paragraph?
- Figures 1-3: Could you add colors to the dots based on language or language family to further illustrate the high diversity of languages?
- The first time you mention "ecological risk" you are referring to it as if the reader knows it already. Please revise.

**Current perspectives**

- The first paragraph is quite unclear. You state there is one question per each dimension, but then all of the singular questions actually consist of two questions. The questions can be formulated into singular questions and the subordinated clauses of "if they could be identified" can be revised as a statement after the questions.
  - I suggest revising these three dimensions into a table, as they are a central part of the MS, and this would make them pop out and easier to reference throughout the MS.
- "I will return to this point later". Unclear. Please indicate in which section you address this
- "Such conditions are said to.." said by whom?

**Language ideologies, diversification trajectories**

- "Nettle himself". Just Nettle will do, no need to specify it further.
- "..the qualitative literature suggests..." which literature? The ones summarized in the paragraph above this one, or something else? Please clarify, and if you are talking about something else, provide references to the literature.
- The sentence starting with "More specifically, in direct...." is very complex and difficult to follow. Not to mention, a one-sentence paragraph. Can you please break it down into smaller sentences and clarify the statement you are making here?
- "The questions are on the table..." Which questions? The ones you mentioned in the beginning of Current Perspectives section? Presumably so, given what follows, but please be clear when you're referring to something explicitly stated in the MS.
- The sentence starting with "It is also in contrast to what we...." is very long. Please break it down to increase clarity.

**Dynamizing linguistic diversity**

- The first sentence of the section is unclear. Origins of what? Which abovementioned perspectives?
- Make the three interrelated points into an actual list to help with clarity. Also avoid complicated sentences when making central statements like the ones you make here.
  - Related to the second point: "how the ranges of languages contract", what do you mean here? Geographical ranges? Geographical contraction? I suggest qualifying the word with geographical or spatial if you are talking about geographical distributions and their changing sizes.

**Spread zones**

- Generally, for this section, why have "residual zone" in the title if you use "accretion zone" in the text instead? Choose the term you use and use throughout.
- "Well-known distinction between spread and accretion zones"? Consider the journal you are writing for, a generalist journal with a wide audience. Even if these concepts are well-known in linguistics, they are not outside of it, and I suggest you open them up for the reader with one-two sentences. To myself, accretion zones are related to tectonic geology, so this sentence is quite confusing to me. Are these concepts used by Nichols (1992; 1997) you reference in the following sentence?
- "The dichotomy references the uneven distribution..." which dichotomy? An expanding and contracting language distribution? If so, then the concepts need to be opened in a more intuitive manner, because "spread zones" imply the geographical ranges of language are expanding. Accretion zones are also implying this (e.g. "quiet accretion of wealth"), but is this just a more slowly growing expansion? If both are expanding, but at different rates, where does the dichotomy come from?
- The paragraph starting with "As far as language geography..." makes several rather strong statements, but has no references to prior work to substantiate the claims. If these are the author's own speculation, it should be stated more clearly. If they are from the work of Nichols (or somebody else), then referring to them appropriately is required.

**How language ranges contract**

- "They also show themselves" is already quite far away to reference the dynamics describing how previously widely spread languages are pushed to the margins. Please revise
- If you are not elaborating on the Vanuatu example by Francois (2012), then I suggest either dropping it or moving it after the Caucasus example. Now it is a bit counter-intuitive to have this as the first example, but no elaboration is provided, while the second example takes up most of the paragraph.

**A new view**

- I agree with the argument made here, but I wish to point out something, that would be good to mention/clarify here, is that languages can not spread without humans, and how humans "spread" is a bit different to species of animals that might have specific requirements for their ecological niche. Throughout the globe, humans have common tendencies in their selection for their habitat and thus, it is fairly logical for the language contraction to start from the most densely populated areas as they have the natural, social and cultural resources to support a larger population. Also, taking the Basque speaker and Roman conquest example: It is also far more logical from an imperial/military perspective to start taking over the towns rather than isolated regions, because these places are more habitable and have more resources than the remote regions. By capturing such places, the invading force takes control of the most important hubs and resources from the defending force, complicating their efforts, and thus causing the original populations having to evacuate to more remote regions with less resources to sustain a larger population. Similar to humans, some animals are generalists and can thrive in a wide variety of environments (seagulls, pigeons, mice, dogs etc) and thus can also retreat to more remote regions as they can survive there as well (likely the Tasmanian tiger was more of a generalist as well). Conversely, other species are highly specialized to specific environmental conditions, and if their habitat is destroyed or another invading species outcompetes them in their ecological niche, they usually die out.

**From empirical case studies**

- The paragraph starting with "While a major issue for the language sciences...".
  - The starting sentence is quite wordy, can you simplify it?
  - The latter half of this paragraph has described very clearly what the authors is going for with the MS, and I think it should be also stated in a succinct manner in the abstract and introduction as well.

Is the argument persuasive and supported by appropriate evidence?

Yes

Is the work clearly and cogently presented?

Partly

Is the topic of the essay discussed accurately in the context of the current literature?

Yes

Does the essay contribute to the cultural, historical, social understanding of the field?

Yes

Reviewer Expertise:

Geography, linguistic landscapes, regional sciences

I confirm that I have read this submission and believe that I have an appropriate level of expertise to confirm that it is of an acceptable scientific standard, however I have significant reservations, as outlined above.

# Reviewer report: Reviewer response for version 2

**DOI:** 10.21956/openreseurope.20844.r49965

**Published:** 2025-04-08

**Authors:** Alexandre François

**Affiliations:** 1 LaTTiCe, Centre National de la Recherche Scientifique, Paris, France; 2 Ecole Normale Superieure, Paris, Île-de-France, France; 3 Linguistics, Australian National University School of Culture History and Language, Canberra, Australian Capital Territory, Australia

This paper is a useful synthesis of various issues raised by the uneven distribution of languages in the geographical space; it brings about an insightful discussion of major concepts discussed in the literature.

 The article covers a broad territory, in all senses of the word, but does so in a generally clear enough manner, that the author’s stream of thought can be followed. This could have been a challenge with such an ambitious paper, considering the array of notions under discussion – such as “language richness”, “phylogenetic diversity”, “structural diversity”, key concepts introduced by Nettle (“ecological risk”, Mean Growing Season), as well as those presented by Nichols, and numerous other authors cited. But the author manages to draw a thread between all these notions, and to make them resonate with each other in a meaningful way.

 Isn’t “language richness” also often labelled “language density” (indeed one of the subtypes “language diversity”)? or are these supposed to be two separate concepts?

 Compared to the earlier version, I believe that the second edition of this paper has gained in clarity – partly due to a neat presentation of concrete examples right from the start. These do help the reader figure out quickly what kind of linguistic diversity is exactly at stake in the paper. The Vanuatu example provides a welcome opportunity to contrast language density (or "richness") with structural diversity, which are respectively high and low here (as rightfully explained p.4). The reader can't help wonder what other combinations  of parameters would be attested around the world. Thus, perhaps an example of a zone with high structural diversity would have been welcome – but I understand this might have made the article too long.

 I also appreciated the discussion comparing linguistic diversity and biodiversity.

 _____

 I will only end with a few notes on harmless errors that can easily be fixed through careful proofreading, in case the author can still edit the text:

 p. 1 *unevently* → *unevenly*

 p. 1 *embarassing* → *embarrassing*

 p. 3 *a slightly situation* → (?)

 p. 4 *ɣilal or ɣilal* → (?)

 p. 4 *jɵjməᶢʟen or jɵjməᶢʟen* → (?)

 p. 4 *addresse *→ *addressee*

 p. 5 *ecotone *→ *ecozone*

 p. 7 *hence, so the hypothesis, fewer *→ (unclear)

 p. 7 *agrictulturalist *→ *agriculturalist*

 p. 7 *quantiative *→ *quantitative*

 p. 9 *instrusive *→ *intrusive*

 p. 9 *characterizes typical characteristcs *→ (?)

 p. 11 *qualitiative *→ *qualitative*

 _____

 Overall, this is an instructive and inspiring paper.

Is the argument persuasive and supported by appropriate evidence?

Yes

Is the work clearly and cogently presented?

Partly

Is the topic of the essay discussed accurately in the context of the current literature?

Yes

Does the essay contribute to the cultural, historical, social understanding of the field?

Yes

Reviewer Expertise:

historical linguistics; linguistic typology; social ecology and language ideologies

I confirm that I have read this submission and believe that I have an appropriate level of expertise to confirm that it is of an acceptable scientific standard.

# Reviewer report: Reviewer response for version 2

**DOI:** 10.21956/openreseurope.20844.r50073

**Published:** 2025-03-26

**Authors:** Hedvig Skirgård

**Affiliations:** 1 Max Planck Institute for Evolutionary Anthropology, Leipzig, Germany

This manuscript presents an overview of several studies pertaining to the unevenness of linguistic diversity and the possible causes thereof, and then presents the author's preferred approach to the field of study. It's overall well-written and well situated in the literature and the proposed approach is sound.

 I do however think that in its current form it is not best serving what I take to be the main aim: presenting a new approach which is process-based, range-focussed and with theoretical foundations in existing qualitative research. There are two main issues as I see it.

 1) Zero in on language richness

 While it's definitely worthwhile to mention other dimensions of language diversity such as phylogenetic diversity and structural diversity, I fear that the space currently given to these in the manuscript distracts from the main message - which seems to me to be the new approach which is primarily (but not only) related to language richness. To streamline the paper, I propose to the author to consider removing some of the content related to other metrics and instead give more space to specifics of the new approach at the end. Alternatively, if the other dimensions of linguistic diversity are kept, there should be more done in the last section to consistently refer back to the implications of the proposed approach on all three mentioned metrics. 

 2) Flesh out the preferred model more

 Currently I am unfortunately not entirely clear on the specifics of the author's recommended approach. For example, are Agent-Based Models something that would fit? More details on the kind of research that the author calls for would be welcome, especially large-scale studies.

 I have also listed other medium and minor points. I am sorry if the review is too long, I am very excited by the topic matter and enjoyed the paper. Please focus on the two main points above, and take only from the rest what you want.

 Medium points:

 I find the authors characterisation of the question of what predicts language richness as "poorly understood" to be too strong. The body of literature that the author cites paints a picture of several relevant predictors, such as certain environmental conditions, political complexity etc. We may not completely understand the distribution of language diversity across the world, but that is not the same as it being "poorly understood". At a simple level, we can for example observe that where there are more people there are more languages. This is why the question of language richness is often linked to general questions about what drives population distributions (e.g. environmental factors like carrying capacity etc). We can see this relationship by taking the number of languages (according to Glottolog 3.0) per country and the population in 1950 per country (according to the UN). A simple Pearson correlation tests gives a moderate significant correlation (R= 0.47, p<0.001); if you log10-transform both variables to account for outliers, the correlation is stronger (R = 0.76, p<0.001). Naturally, this isn't enough analysis by far (countries are not good units, we'd want older population data, more sophisticated statistical models, correlation is not causation etc), but I think it illustrates that population size is probably one of several relevant parameters and that we can look more broadly at literature that studies the uneven distribution of people over the world.

 On a related note, the link between number of languages and number of people becomes trickier when we consider that cultures with high political complexity and agriculture tend to have larger populations and spread over larger areas (c.f. Currie & Mace 2009) and hence reduce the language richness of their region. I'd appreciate the author's insights into how to approach the effect of population size on language richness in relation to spread and accretion zones. Is it the case that there's a minimal population that is needed for language diversity, but that once that limit is reached other factors are more important?

 Regarding accretion zones, the author may be interested to read Graff et al (2024, pre-print) where they study the interaction between genetic and language diversity, finding that regions with high linguistic diversity feature low genetic diversity which suggests more relative isolation.

 Page 3: "sub-saharan Africa" is a huge region which contains many families besides Bantu (and even in addition to the larger family of Atlantic-Congo to which the Bantu languages belong). There's Khoisan, Mande, Khoe-Kwadi, Afro-Asiatic; not to mention language isolates and other families. For an example of a place with high language richness and uncontroversially low phylogenetic diversity, I'd suggest sticking with Vanuatu where the field agrees that all indigenous pre-contact languages are Oceanic.

 Page 4, footnote 1: as the author has chosen to only plot languoids which are of the level "language" in Glottolog, I think the discussion of the term "languoids"  is superfluous. 

 I second Dr Inglese that a working definition of a language is warranted. Instead of discussing languoids, I propose that the author refers to Glottolog's definition of "language" (Hammarström et al 2020), Gooskens & Schneider's work (i.a. 2016) and Ethnologue's description of their process (Eberhard et al 2019).

 Page 11: I appreciate the author's use of the work by Pacheco Coelho et al (2019). One of the possible concerns with non-stationary effects is the potential for over-fitting models. Pacheco Coelho et al (2019) guard against this by for example spatial smoothing, not allowing estimates to fluctuate too much between adjacent regions. Does the author have any comments on how their overall approach would be sensitive to overfitting and if so, how this could be mitigated?

 Page 11: the reference to a "brute evolutionary view" came a bit out of left field for me and felt needlessly harsh. I'm not sure I understand what is being referred to here. I found this characterisation a bit too strong and would recommend a more specific description instead.

 Minor points: 

 Page 3 "a slightly situation" -> "a somewhat similar situation" or other phrase

 Page 5: language richness and language density are used essentially interchangeably. To make it easier on the reader, please consider using "language richness" only.

 Page 7: if possible, I'd recommend avoiding the term "Melanesia" as it is not always particularly helpful for an academic historical perspective. "Melanesia" encompasses a very wide diversity of communities and is not scholarly defined. I believe that Sankoff (1980) mainly discusses parts of Papua New Guinea specifically. Laycock (1982) does discuss Melanesia more broadly, but mainly exemplifies with Papua New Guinea and New Caledonia. If there is such a thing as a specific cultural ideology that is truly well-spread over the entire region and emblematic of it such that it is not found elsewhere, I am not convinced the current citations are enough. For the argument in this paper, I believe the Melanesian framing can be dropped and the author can go straight to the example and specifically cite work on Vanuatu (and/or Australia). 

 Page 7: reference addition suggestions, the earliest paper I know for "egalitarian bi/multilingualism" is Haudricourt (1961) and one of the more recent overviews is Vaughan & Singer (2018). Evans (2018:21) discusses more places in which egalitarian multilingualism has been documented. Haudricourt (1961) also discuses Caucasus, so if the author wants to reference the earliest source that could be done in sections relation to Caucasus.

 Page 7: The author refers to Thurston (1989), Trudgill (2011) etc. If the author wants to delve further into the literature on social ecology of languages, I've included at the end a list of terms and citations that I personally keep on hand in order to distinguish different related concepts in the literature. In particular, I believe it could be advantageous to discuss linguistic epidemiology/propagation in relation to esotereogny/hyperdialectism and the situation described by Nichols (2013) in the Caucasus. However, I'm also sensitive to the length of the essay already so this can also be ignored in favour of brevity.

 Page 9: accretion and residual zone are used interchangeably, please do mention both the first time but after that just use one (I prefer accretion).

 ________________________________________

 Terminology list mentioned earlier

 * metatypy 

 [a] change in morphosyntactic type and grammatical organisation [and also semantic patterns] which a language undergoes as a result of its speakers’ bilingualism in another language. This change is driven by grammatical calquing, i.e. the copying of constructional meanings from the modified language and the innovation of new structures using inherited material to express them.

 Ross (1999); Gumperz & Wilson (1971); Kulkarni-Joshi (2016)

 * calquing

 the borrowing of structural content from another language which increases inter-translatability (one-to-one mappings)

 * esoterogeny

 The process of making a language more esoteric and thus more difficult for non-speakers to understand

 Thurston (1987)

 * schismogenesis

 a process of differentiation in the norms of individual behaviour resulting from cumulative interaction between individuals

 Bateson (1936)

 * neighbour- opposition

  when one finds forms in a language variety which are not predictable historically from Neogrammarian sound laws and which function to make the distance from a neighbouring variety greater than would otherwise have been the case [...]The linguistic feature in question is often seen by the speakers as a salient and even, in some cases, symbolic or shibboleth feature of that variety.

 Larsen (1886); Jahr (2017)

 * hyperdialectism

 when dialect speakers overgeneralise differences [..] in order to symbolise their separate identities

 Trudgill (2003)

 * linguistic epidemiology / propagation

 strong convergence within groups which has the by-effect of divergence between groups

 Enfield (2003, 2008); Toulmin (2009)

 * doppel-avoidance

 bilinguals avoiding potential false-friends by choosing diverging lexical items

 Ellison & Miceli (2017)

 ________________________________________

 References:

1. Bateson, G. (1936) [Ref 1]
2. Currie TE, et al., 2009 [Ref 2]
3. Eberhard, D. M., Simons, G. F., & Fennig, C. D. (2019). The problem of language identification. In D. M. Eberhard, G. F. Simons, & C. D. Fennig (Eds.), Ethnologue: Languages of the World (22nd ed.). Dallas, Texas: SIL International. https://www.ethnologue.com/about/problem-language-identification.
4. Ellison, T. M. et al., 2017 [Ref 3]
5. Enfield, N. 2013 [Ref 4]
6. Enfield, N. 2008 [Ref 5]
7. Evans, N. (2018). The dynamics of language diversity. In R. Mesthrie & D. Bradley (Eds.), The dynamics of language: Plenary and focus lectures from the 20th International Congress of Linguists (pp. 12–35). Claremont: UCT Press. https://ciplnet.com/wp-content/uploads/2024/02/ICl20_Plenary-and-focus-lectures.pdf 
8. Gooskens, C., & Schneider, C. (2016). Testing mutual intelligibility between closely related languages in an oral society. https://hdl.handle.net/10125/24696
9. Graff, A., et al., 2024 [Ref 6]
10. Gumperz, J. J., & Wilson, R. (1971). Convergence and creolization. Pidginization and Creolization of Languages, 151, 167. https://search.worldcat.org/title/1311039317
11. Hammarström, H.,et al., 2019 [Ref 7]
12. Haudricourt, A. 1961 [Ref 8]
13. Jahr, E. H. 2017 [Ref 9]
14. Kulkarni-Joshi, S. 2016 [Ref 10]
15. Larsen, A. B. (1886). Oversigt over de trondhjemske dialekters slægtskabsforhold [Overview of the relationships between the dialects of Trøndelag]. In Det Kongelige Norske Videnskabers Selskabs Skrifter 1885 (pp. 37–117). Trondheim, Norway: Interessentsskabets Bogtrykkeri. https://search.worldcat.org/title/10348260381
16. Laycock, D. C. (1982). Melanesian linguistic diversity: A Melanesian choice? In R. J. May & H. Nelson (Eds.), Melanesia: Beyond diversity. https://openresearch-repository.anu.edu.au/bitstream/1885/133344/1/RSPAS_Melanesia_Beyond_Diversity.pdf
17. Maclaurin, J, et al., 2008 [Ref 11]
18. Pacheco Coelho, et al., 2019 (Ref 12)
19. Ross, M. (1999). Exploring metatypy: How does contact-induced typological change come about? Keynote talk given at the Australian Linguistic Society’s annual meeting, Perth. https://www.academia.edu/3404466/Exploring_metatypy_how_does_contact_induced_typological_change_come_about
20. Thurston, W. R. (1987). Processes of change in the languages of North-Western New Britain. Canberra: Pacific Linguistics. https://openresearch-repository.anu.edu.au/bitstream/1885/145417/1/PL-B99.pdf
21. Vaughan, J, et al., 2018 [Ref 13]

Is the argument persuasive and supported by appropriate evidence?

Yes

Is the work clearly and cogently presented?

Partly

Is the topic of the essay discussed accurately in the context of the current literature?

Yes

Does the essay contribute to the cultural, historical, social understanding of the field?

Yes

Reviewer Expertise:

linguistic typology, cultural evolution, historical linguistics

I confirm that I have read this submission and believe that I have an appropriate level of expertise to confirm that it is of an acceptable scientific standard, however I have significant reservations, as outlined above.

## References

[1] : Naven.1958; 10.1515/9781503621138 10.1515/9781503621138

[2] : Political complexity predicts the spread of ethnolinguistic groups. *Proc Natl Acad Sci U S A* .2009;106(18) : 10.1073/pnas.0804698106 7339-44 10.1073/pnas.0804698106 19380740 PMC2670878

[3] : Language monitoring in bilinguals as a mechanism for rapid lexical divergence. *Language* .2017;93(2) : 10.1353/lan.2017.0014 255-287 10.1353/lan.2017.0014

[4] : Linguistic Epidemiology.2013; 10.4324/9780203605196 10.4324/9780203605196

[5] : Transmission Biases in Linguistic Epidemiology. *Journal of Language Contact* .2008;2(1) : 10.1163/000000008792525273 299-310 10.1163/000000008792525273

[6] : An inverse correlation between linguistic and genetic diversity. *bioRxiv* .2024; 10.1101/2024.12.18.628602 10.1101/2024.12.18.628602

[7] : glottolog/glottolog: Glottolog database 4.0. *https://zenodo.org/records/3260726* .2019; 10.5281/zenodo.3260726 10.5281/zenodo.3260726

[8] : Richesse en phonèmes et richesse en locuteurs. *L'Homme* .1961;1(1) : 10.3406/hom.1961.366337 5-10 10.3406/hom.1961.366337

[9] : A pioneering sociolinguistic concept: Amund B. Larsen and his discovery in the 1880s of neighbour opposition as a socio-psychological mechanism in linguistic change – and its rediscovery in the 1980s as hyperdialectism. *European Journal of Scandinavian Studies* .2017;47(2) : 10.1515/ejss-2017-0020 10.1515/ejss-2017-0020

[10] : Forty years of language contact and change in Kupwar: A critical assessment of the intertranslatability model. *Journal of South Asian Languages and Linguistics* .2016;3(2) : 10.1515/jsall-2016-0008 147-174 10.1515/jsall-2016-0008

[11] : What Is Biodiversity?.2008; 10.7208/chicago/9780226500829.001.0001 10.7208/chicago/9780226500829.001.0001

[12] : Drivers of geographical patterns of North American language diversity. *Proc Biol Sci* .2019;286(1899) : 10.1098/rspb.2019.0242 20190242 10.1098/rspb.2019.0242 30914010 PMC6452074

[13] : Indigenous multilingualisms past and present. *Language & Communication* .2018;62: 10.1016/j.langcom.2018.06.003 83-90 10.1016/j.langcom.2018.06.003

# Response

**Published:** 2025-04-13

**Authors:** Matthias Urban

Thank you very much for your careful read and detailed comments!

 In response to the main points, I've revised the manuscript as follows:

 to address the imbalance between more review-oriented text with all three dimensions of diversity in scope on the one hand and relative brevity of the different kind of approach I have in mind, I've basically shortened the former and focused more (though not exclusively) on language richness, and expanded discussion of the latter.

 In particular, the section titled "Dynamizing linguistic diversity" now contains subsections (as requested by reviewer #4). These include a subsection titled "From empirical case studies of range reduction to generalizations on linguistic diversity" that contains entirely new text and describes ways to approach linguistic diversity based on the dynamics of language expansion and range reduction. I've also added text on the comparison of typological distributions this will enable to "do something" with that parameter later on in the text as well. I've deliberately kept the discussion conceptual and non-technical (though I mention summary statistics on polygons as a way to quantify environment) ... I'm thinking of this as a conceptual essay and operationalization etc. would be a matter for subsequent papers. 

 Thank you also for the helpful "meso" and "minor" comments. I've made good use of most, though not all. For example, since this is really a programmatic essay, I would like to keep it non-technical and not go into modeling issues like overfitting when allong nonstationary effects or factoring in population size, and I think I'd like to keep the "languoid" in - it's only in a footnote, and it does justice to the difficulty with languages as reified notions.

# Reviewer report: Reviewer response for version 2

**DOI:** 10.21956/openreseurope.20844.r49963

**Published:** 2025-01-22

**Authors:** Guglielmo Inglese

**Affiliations:** 1 Universita degli Studi di Torino, Turin, Piedmont, Italy

I am pleased with the revised version of the paper and have no further comments to make.

Is the argument persuasive and supported by appropriate evidence?

Yes

Is the work clearly and cogently presented?

Yes

Is the topic of the essay discussed accurately in the context of the current literature?

Yes

Does the essay contribute to the cultural, historical, social understanding of the field?

Yes

Reviewer Expertise:

historical linguistics and linguistic typology

I confirm that I have read this submission and believe that I have an appropriate level of expertise to confirm that it is of an acceptable scientific standard.

# Reviewer report: Reviewer response for version 1

**DOI:** 10.21956/openreseurope.19907.r46601

**Published:** 2024-12-30

**Authors:** Tuomas Väisänen

**Affiliations:** 1 Department of Geosciences and Geography, University of Helsinki, Helsinki, Uusimaa, Finland; 2 Helsinki Institute of Sustainability Science, University of Helsinki, Helsinki, Uusimaa, Finland; 3 Helsinki Institute of Urban Regional Studies, University of Helsinki, Helsinki, Uusimaa, Finland

I welcome and enjoy any manuscript, such as this one, that bridges the gap between geography and linguistics. The article describes how linguistic diversity and its geographical distribution have been studied in the field of linguistics over the past few decades, and proposes combining several theories (climatic, environmental, economic, language ideological) that attempt to explain why the diversity of languages varies geographically across the globe. The author manages to make a convincing case for his proposal, while pointing out remaining areas which still need to be worked on. In summary, I quite like the manuscript and find it makes a valuable contribution.

 However, I have two minor conceptual issues and one minor structural issue. The remaining comments are even more minor technical stuff regarding typographical mistakes and textual clarity. First, I find it a bit confusing the author uses both "linguistic diversity" and "language diversity" while ostensibly referring to the same phenomena. However, if they are different, the distinction between these two concepts should be explained to the reader clearly. Now, it is not mentioned in the manuscript. Second, the author does not state which type of "linguistic diversity" he means, which is puzzling as the introduction lays out the different meanings the concept has (number of languages, number of language families, structural diversity). This is crucial information as it is the central concept, and it is operationalized throughout the manuscript. My take after reading the manuscript is that the author is mostly talking about the number of different languages, unless explicitly mentioning language families, but this should be explicitly stated. Third, the "Current perspectives" and "Dynamizing linguistic diversity" sections would benefit from sub-sectioning (and potentially some minor restructuring) as they are long and the reader has forgotten the three main perspectives or points by the end of the section.

 Overall, the article is already in rather good shape, and my recommendation is a minor revision for the above-mentioned reasons. Although, I must confess that I am a geographer first and foremost, so I am leaning on the other reviewers' remarks on the author's grasp on the theoretical literature on historical linguistics.

 Below are my detailed comments:

**Introduction**

- I have a comment on (or perhaps a slight issue with) the author's delineation of three types of understanding "language diversity". I argue there is a fourth type of language diversity, that draws on methods commonly used in ecology to quantify the diversity of species (see work of Peukert, 2013 [Ref 1]; Hiippala T, et al., 2019 [Ref 2]; Hiippala T, et al., 2020[Ref 3]). In fact, I think this fourth type could be integrated with the first or second examples provided (the "language richness" and "language family richness" examples), as it is related to quantification of linguistic diversity (that is, the diversity of languages). However, this additional understanding of language diversity goes a bit further than just the sheer number of languages in a given study area, and is focused on several perspectives simultaneously: richness, dominance, evenness, and diversity.
  - These perspectives can be quantified (or estimated) by a wide selection of metrics, each with their own benefits and drawbacks. In this context, richness is in its simplest form the number of languages, as the author states, but not only that. Dominance describes whether the given sample is dominated by one or more languages. Evenness describes the between-class similarity of the distribution of languages, that is, if all languages are spoken by as many individuals. Diversity tries to incorporate both richness and evenness into one metric.
- Some unclear sentences and unnecessary self-referential language in the paragraph discussing Figure 3 (starting p3)
- Last paragraph of Introduction on page 5. Please provide a reference to this "particular interpretation of a prominent hypothesis". Furthermore, I don't see how the starting sentence of this paragraph is connected to the rest of the sentences in the paragraph.

**Current perspectives**

- General comment: I suggest some slight restructuring and adding subsections that correspond to the three dimensions (or are they perspectives, as the section title leads the reader to believe?) laid out here. This would help the reader follow the arguments made better.
- In the starting paragraph: what is/are "lowbeds linguistic landscapes"?
- Can there be one sentence paragraphs in an ORE article? I am referring to the paragraph starting with "Early studies (Cashdan, 2001....". If not, I would integrate this somewhere else or flesh it out.
- The sentence "As a whole, research, reassuringly, shows the typical signs of maturation of a field of scientific investigation". Please rephrase this sentence. I take it, you mean this specific subfield of research has matured, but now you're referring to research in general. A similar thing happens in the last paragraph of the "Current perspectives" section in the beginning of the paragraph.
- The use of "largest imaginable scale" is a bit vague and over the top here. I presume you mean "globally", please clarify.
- Please rephrase the last sentence of this paragraph, it is very long and difficult to understand.

**Dynamizing linguistic diversity**

- General comment on this section: I think this section would improve if you could divide it into subsections that relate to the three interrelated points you want to make, especially as it is the section containing the main contribution by the author's own words. Now, it is a bit hard to follow how you address these points.
- Last sentence of the first paragraph. Please elaborate how your approach departs from the case studies.
- Paragraph starting with "Nichols also characterizes…" on page 8 requires a reference to the work(s) of Nichols being referred to here.
- On page 9, there is a partially missing subordinate clause in the sentence starting with "As a result, the oldest layers of the diverse linguistic landscape..."

**Conclusion**

- The first sentence is very long. Please consider dividing it into two or more sentences.
- Another paragraph of only one sentence here. Please flesh it out, or integrate somewhere else.
- The last sentence has a missing space between words. 

Is the argument persuasive and supported by appropriate evidence?

Yes

Is the work clearly and cogently presented?

Partly

Is the topic of the essay discussed accurately in the context of the current literature?

Yes

Does the essay contribute to the cultural, historical, social understanding of the field?

Yes

Reviewer Expertise:

Urban geography, linguistic diversity, geoinformatics, regional science

I confirm that I have read this submission and believe that I have an appropriate level of expertise to confirm that it is of an acceptable scientific standard, however I have significant reservations, as outlined above.

## References

[1] : Measuring language diversity in urban ecosystems.2: 10.1075/hsld.2.06peu 75-96 10.1075/hsld.2.06peu

[2] : Exploring the linguistic landscape of geotagged social media content in urban environments. *Digital Scholarship in the Humanities* .2019;34(2) : 10.1093/llc/fqy049 290-309 10.1093/llc/fqy049

[3] : Mapping the languages of Twitter in Finland:. *Neuphilologische Mitteilungen* .2020;121(1) : 10.51814/nm.99996 12-44 10.51814/nm.99996

# Response

**Published:** 2025-04-13

**Authors:** Matthias Urban

Thank you very much for your comments, and apologies for the long time it has taken me to respond. Your comments were received almost exactly at the point I had prepared a revision based on the previously received three reports so I thought it wiser to wait with a further round of revision until all reports are in.

 I've revised the text taking into account both your main points of criticism as well as the minor ones. Concerning the former, I've replaced "language diversity" with "linguistic diversity" except in one direct quote - no difference is implied by the terms. I have made clear that indeed language richness is the main variable I look at in the introduction, though in response to one of the main points of reviewer

 #5 I've included some more discussion of typological diversity to the section "Dynamizing linguistic diversity". At any rate, it should be clearer now what I am talking about in each particular instance. I've also added subsections to the main sections to guide the reader better. Finally, I've taken care of the further comments you made on individual sections (some were superseded already in the first revision).

# Reviewer report: Reviewer response for version 1

**DOI:** 10.21956/openreseurope.19907.r45031

**Published:** 2024-12-02

**Authors:** Alexandre François

**Affiliations:** 1 LaTTiCe, Centre National de la Recherche Scientifique, Paris, France; 2 Ecole Normale Superieure, Paris, Île-de-France, France; 3 Linguistics, Australian National University School of Culture History and Language, Canberra, Australian Capital Territory, Australia

The paper “Global language geography and language history: challenges and opportunities” raises the question of linguistic diversity and of the uneven distribution of languages around the world. It discusses what different factors can account for it – including geographical, ecological, economical, ideological factors. This text is rather rich in references, taken from different disciplines relevant to this discussion. Its argument is sound and convincing.

 The section on geographical and ecological factors – around the notion of “ecological risk” – is insufficiently clear about its subject. We understand that access to food and resources is an important key, but key to what exactly? to increasing human density in general (regardless of how many languages are spoken)? to favouring more numerous, smaller-size communities? Do these economic factors favour linguistic *density* (number of languages per capita)? or do they go as far as fostering linguistic *diversity* strictly speaking – i.e. the structural distance between neighbouring languages? I wish the author brought more clarity to the discussion, so the reader can see exactly what would be direct causes vs. indirect factors explaining language diversity.

 The discussion about language ideologies would gain from incorporating the notion of “egalitarian multilingualism”: this attitude among neighbouring groups has been described in the literature (e.g. on Vanuatu, and Melanesia more generally) as an important factor in the maintenance or even the increase of linguistic diversity. In many parts of the world, language is taken as emblematic of place, an attitude which has been shown to intensify the divergence of languages over time. This results in a social ecology where smaller language communities are favoured, bringing about a tightly fragmented linguistic landscape (cf. studies on Vanuatu, or on Australia).

 The passage on spread vs. residual zones is an excellent synthesis of J. Nichols’s reflection on different language ecologies.

 On the formal side, the current version of the text still has many typos, as well as some stylistic imperfections (excessive hypotaxis; repetitions; some unclear passages), and minor errors (e.g. "ephemeral" instead of "epiphenomenal"; "southwesternmost" for "southeasternmost"…) Yet this could be easily corrected by the author.

 Overall, this paper is a solid synthesis of the last decades of reflection on an important topic, namely the factors underlying the distribution of languages around the planet. While it says little about the languages themselves (e.g. which aspects tend to change vs. which tend to be stable over time?), it does bring together the viewpoints of various disciplines, to construct an inspired reflection that bridges linguistics and human geography.

Is the argument persuasive and supported by appropriate evidence?

Yes

Is the work clearly and cogently presented?

Partly

Is the topic of the essay discussed accurately in the context of the current literature?

Yes

Does the essay contribute to the cultural, historical, social understanding of the field?

Yes

Reviewer Expertise:

historical linguistics; linguistic typology; social ecology and language ideologies

I confirm that I have read this submission and believe that I have an appropriate level of expertise to confirm that it is of an acceptable scientific standard, however I have significant reservations, as outlined above.

# Response

**Published:** 2024-12-22

**Authors:** Matthias Urban

Thank you for reading the article and for your extensive and thoughtful comments! I have taken them into account in revising the manuscript. I have clarified that indeed language density is what is thought to depend on "ecological risk", this indeed wasn't fully clear. You will note that I make quite extensive reference to Vanuatu with reference to your work in the revised version. This serves multiple purposes: on the one hand, it provides an opportunity for actual linguistic exemplification requested by reviewer #1; it provides an opportunity to give an example of a local language ecology and its linguistic outcomes; and it provides an opportunity to introduce "egalitarian multilingualism", as you request. Finally, I have rewritten the text relatively extensively to make it less prolix and easier to follow the line of thought, and I have emended the wrong choices of words you noted (thank you for your close reading!).   

# Reviewer report: Reviewer response for version 1

**DOI:** 10.21956/openreseurope.19907.r45382

**Published:** 2024-11-28

**Authors:** Guglielmo Inglese

**Affiliations:** 1 Universita degli Studi di Torino, Turin, Piedmont, Italy

The paper offers a fresh and stimulating overview current approaches to explaining linguistic diversity and the distribution of languages worldwide. It explores a number of shortcomings in existing research that links linguistic diversity to environmental factors and proposes, as an alternative, a focus on the historical process of language spread and recess that result in synchronic language diversity patterns. The paper is overall well written and provides an exhaistive and informed survey of the topic, while also laying a clear ground for future research. Its argumentation is nicely supported by a number of specific examples (e.g. the situation of Basque and that of the languages of the Caucasus). I have a few suggestions on points that deserve furth er improvement. First, a definition of what counts as a language is missing: this is both an empirical issue (in the sense that is not clear on which data figures 1 to 3 are based) as well as a more theoretical one. Even a working definition, such as that provided by Gil 2016 (but other definitions may be good as well, this is just one example), could make the point clearer. Second, it might be worth stressing that a shift in focus to processes in explaining linguistic diversity finds a parellel in current trends in source-oriented typology (I'm thinking of a number of papers by Sonia Cristofaro, e.g. Cristofaro 2019 (Ref 3) among others). These represent different ways in which historical thinking may reshape our understanding of linguistic diversity. Finally, the brief mention of the connection between the study of worldwide linguistic diversity with dialectology/variationist sociolinguistics might be expanded upon (see the classic Trudgill 2011 and the discussion in Inglese & Ballarè 2023).

Is the argument persuasive and supported by appropriate evidence?

Yes

Is the work clearly and cogently presented?

Yes

Is the topic of the essay discussed accurately in the context of the current literature?

Yes

Does the essay contribute to the cultural, historical, social understanding of the field?

Yes

Reviewer Expertise:

historical linguistics and linguistic typology

I confirm that I have read this submission and believe that I have an appropriate level of expertise to confirm that it is of an acceptable scientific standard, however I have significant reservations, as outlined above.

## References

[1] : 1 Analyzing language variation: Where sociolinguistics and linguistic typology meet.2023; 10.1515/9783110781168-001 1-28 10.1515/9783110781168-001

[2] : Describing languoids: When incommensurability meets the language-dialect continuum. *Linguistic Typology* .2016;20(2) : 10.1515/lingty-2016-0017 439-462 10.1515/lingty-2016-0017

[3] : Taking diachronic evidence seriously: Result-oriented vs. source-oriented explanations of typological universals. *https://zenodo.org/records/2583806* .2019; 10.5281/zenodo.2583806 10.5281/zenodo.2583806

# Response

**Published:** 2024-12-22

**Authors:** Matthias Urban

Thank you for reading and commenting on the article! I have added relatively extensive discussion of where the data come from (viz. Glottolog) and the policies for assignment of coordinates. Extended discussion of the notion of "languoid" that is relevant here as well is in note [1]. A reference to "sociolinguistic typology" à la Trudgill is added to the main text, and a reference to dynamic diachronic approaches to typology in note [3].

# Reviewer report: Reviewer response for version 1

**DOI:** 10.21956/openreseurope.19907.r45380

**Published:** 2024-11-05

**Authors:** Stan Brunn

**Affiliations:** 1 University of Kentucky, Lexington, Kentucky, USA

An original, insightful and well referenced and written statement exploring linguistic diversity in new contexts: environmental and climate variable AS WELL as different continental and regional settings. I would ask the author to address mapping the location of some groups to show the difficulties in mapping the "degrees" of diversity in some historical and contemporary contexts.  This focus would again address the problems of not only mapping "where a language is spoken" but also how "slippery" it is to map locations across a linguistic space, but also over time. Second, the paper could/would be stronger IF some examples of words or phrases were used by certain groups over time or in a place.  A most thought-provoking contribution.

Is the argument persuasive and supported by appropriate evidence?

Yes

Is the work clearly and cogently presented?

No

Is the topic of the essay discussed accurately in the context of the current literature?

Yes

Does the essay contribute to the cultural, historical, social understanding of the field?

Yes

Reviewer Expertise:

the intersections of social, cultural, political and environmental geographies.

I confirm that I have read this submission and believe that I have an appropriate level of expertise to confirm that it is of an acceptable scientific standard.

# Response

**Published:** 2024-12-22

**Authors:** Matthias Urban

Thank you for reading and commenting on this article. I have included the additional information you requested in the revised version, including sample linguistic data from Vanuatu that illustrate different aspects of linguistic diversity
